# How to design subsidy policies to better encourage travelers to use car-sharing instead of private cars? An evolutionary game study

**DOI:** 10.1371/journal.pone.0308622

**Published:** 2024-09-19

**Authors:** Zixun Li, Yue Sun, Gang Zong, Xianlei Dong

**Affiliations:** 1 School of Business, Shandong Normal University, Jinan, Shandong, China; 2 School of Economics, Beijing Technology and Business University, Beijing, Beijing, China; 3 School of Economics and Management, Dalian University of Technology, Dalian, Liaoning, China; 4 School of Economics and Management, Beijing University of Technology, Beijing, Beijing, China; The University of Texas at El Paso, UNITED STATES OF AMERICA

## Abstract

Car-sharing is a travel mode that can serve as an alternative to private cars, helping to reduce urban pollution. However, currently, there is a low willingness among travelers to use car-sharing, which is reflected in both low market penetration and user frequency. Therefore, it is essential for the government to encourage the use of car-sharing by providing subsidies. To better encourage the usage of car-sharing, this paper applies a two-fold evolutionary game model involving travelers and the government to explore the impact of subsidies on travelers’ choices, and the factors that could affect the subsidies’ efficiency. A simulation, using data from Beijing, was conducted to determine the implications of subsidy policies. The results show that a mileage-based subsidy and a fixed subsidy are applicable to travel of high and low mileages respectively, and under both subsidy modes, subsidies for trips with short duration or short pick-up and return time are more effective. Furthermore, we find that the efficiency of subsidies increases as the scale of car-sharing users, demand elasticity, or total number of travelers increases. Additionally, the subsidy levels should be lower than the environmental benefits of car-sharing but higher than the difference in travel costs between private cars and car-sharing. Future work will involve other game players such as car-sharing operators in order to draw deeper conclusions, and will involve the collection of data from more countries and cities to develop the robustness of the conclusions.

## 1. Introduction

In recent decades, with the growth of the global economy, the number of private cars in various countries has risen significantly. For instance, data reveals that the number of private cars in China has increased from 2.5 million in 1990 to 260 million in 2022 [1]. However, this rapid surge in the ownership of private cars has also led to severe pollution due to the emission of greenhouse gases and particulate matter [2]. In France, private cars account for over 16% of carbon dioxide emissions [3]. One way to alleviate this issue is through car-sharing [4]. Car-sharing refers to a service in which the operator provides vehicles for travelers, who drive the vehicles themselves and pay vehicle-usage fees based on travel time and mileage. In addition, travelers pick vehicles up and return them from and to designated stations [5, 6]. Presently, car-sharing operators with a large market share include Zipcar, Gofun and others. The operation and management of car-sharing are handled by the operators, bringing significant convenience to travelers. Specifically, operators must expand their fleet of vehicles, and offer a variety of vehicle types, to meet as many travel needs as possible [7–9]. They also need to maintain the vehicles, solve any vehicle technical issues, and supply energy to ensure the vehicles’ normal operation [7], saving the travelers from having to spend time on such tasks. Car-sharing is considered an important alternative to the private car, and plays a crucial role in mitigating gas emissions [5]. Firstly, compared with private cars, car-sharing operators often adopt more fuel-efficient vehicles [10]. For example, in China, new-energy vehicles account for over 90% of the car-sharing fleet [6]. Secondly, research across multiple countries indicates that, on average, one shared car replaces 4 to 23 private cars on the road [11–14]. This reduction in vehicle numbers leads to decreased emissions. Additionally, the diminished demand for vehicles contributes to lower auto production and, consequently, reduced emissions during manufacturing [13]. Thirdly, transitioning from private to shared cars prompts travelers to modify their travel habits. They tend to decrease their travel mileage, thereby lowering emissions [15].

However, the replacement of private cars with car-sharing still has a long way to go. One significant issue lies in the insufficient willingness of travelers to use car-sharing. This is specifically reflected in two aspects: Firstly, there is the low market penetration rate of car-sharing [2], which means that few travelers who have the capability to use car-sharing actually select to use it. According to the Statistics [16], as of 2024, the average global market penetration rate for car-sharing is only 0.8%. Secondly, users who have tried car-sharing often use it only occasionally rather than as a long-term replacement for private cars [17]. According to a survey in Latvia in 2021, 51% of users traveled by car-sharing less than once a month [18], and in Germany, in 2022, 78.3% of users traveled by car-sharing less than once a week [19]. Other countries, like China, also show a low user frequency. According to one study, over 80% of China users of car-sharing services used them less than once a month in 2020 [20].

Car-sharing, as discussed, can not only meet travel demand but also help alleviate pollution, demonstrating positive externalities. Therefore, in accordance with the principle of Pigouvian taxation, the government can implement subsidy policies to encourage travelers to replace their use of private cars with car-sharing [21]. Some countries have already begun incentivizing car-sharing usage through subsidy policies. For instance, the French government will provide a subsidy of 100 euros to each car-sharing user from 2023 [22]. It is likely that more countries will consider implementing subsidy policies in the future. While a subsidy is a useful approach to achieving a particular goal, it also comes with a high cost. Therefore, it is crucial to explore the design of subsidy policies that can better incentivize travelers to use car-sharing. This paper aims to conduct such an exploration.

Several studies have already delved into the topic of government subsidies for car-sharing users, especially exploring the influence of subsidies on users’ willingness to adopt car-sharing [2]. These studies have examined various aspects, such as subsidy amounts and recipients, and their impact on the subsidy’s efficiency [23–25]. While these studies have yielded some valuable insights, there are still areas that require further exploration. Firstly, previous studies have often overlooked the consideration of subsidy costs. In reality, governments may be concerned about this. An abrupt termination of subsidies by the government could have a significant impact on the market, as exemplified by the rapid contraction of China’s electric vehicle market after government subsidies were withdrawn [26]. Therefore, it is essential to focus on the sustainability of subsidy policies. Secondly, previous studies have not considered the influence of car-sharing market conditions (such as market size) and users’ travel characteristics (such as travel mileage) on the efficiency of subsidies. These factors could potentially distort the impact of subsidies [27, 28], leading to inefficient subsidy allocation. To fill these gaps, this study, by drawing on evolutionary game theory, establishes a two-fold evolutionary game model that involves both the government and travelers. This model incorporates market conditions and travel characteristics as influencing factors. Using data from Beijing in 2022, this study simulates the strategic evolution of both travelers and the government when varying amounts of government subsidies are applied, to explore the impact and sustainability of subsidy policies. Then, simulations are conducted to observe the strategic evolution of travelers when adjusting the values of variables related to market conditions and travel characteristics under fixed subsidy and mileage-based subsidy modes, so that these variables’ impact on the efficiency of subsidy policies can be analyzed. Finally, this paper provides some principles for designing subsidy policies based on these simulations.

Compared to previous studies, this study makes the following contributions. In terms of ideas, few previous studies focus on subsidy costs. This paper’s discussion of subsidy policies is based on their sustainability, that is, considering the subsidy costs in the model, thereby enhancing their feasibility and aligning them more closely with reality. Additionally, this paper analyzes the influence of variables related to market conditions and travel characteristics, such as demand elasticity and travel mileage, on subsidy efficiency. Previous studies have mainly focused on the costs of traveling by car-sharing or private car, such as service fees and fuel costs [24]. Furthermore, this paper compares different user subsidy models, which little research has done before now. Therefore, this paper is expected to draw some new conclusions and provide richer and more targeted suggestions for the design of subsidy policies.

In terms of results, firstly, many studies have found that car-sharing has cost advantages for low-mileage travel, making it suitable for short trips. This paper finds that, with mileage-based subsidies, car-sharing has cost advantages for longer trips and these subsidies are sustainable, which would make it possible to encourage the use of car-sharing for high-mileage travel and promote its wider adoption. Secondly, previous research has not thoroughly explored the applicable subsidy levels for car-sharing users, while this paper provides suggestions for setting subsidy levels. Specifically, the subsidy amount should be lower than the environmental benefit of car-sharing but higher than the difference in travel costs between private cars and car-sharing. Thirdly, existing literature has rarely focused on the influence of market conditions on subsidy policies. This paper finds that applying subsidy policies to regions with higher market potential and higher demand elasticity of car-sharing leads to better subsidy efficiency. This finding provides practical recommendations for subsidy policy design and the improvement of subsidy efficiency.

The remainder of this paper is organized as follows: Section 2 reviews related studies; Section 3 constructs an evolutionary game model; Section 4 conducts numerical simulations; Section 5 discusses the simulation results; Section 6 gives conclusions and ideas for future work.

## 2. Literature review

### 2.1 Government subsidies for car-sharing

Government subsidies for car-sharing include two aspects: those provided to car-sharing service providers and those to car-sharing users. In this section, we review the literature on both aspects. Subsequently, we evaluate the impact of government subsidies on the transportation system’s efficiency and review the factors that influence the adoption of car-sharing.

#### 2.1.1 Government subsidies to car-sharing service providers

Government subsidies to service providers are primarily made for shared infrastructure and vehicle purchases. For example, the Korean government offers service providers public parking spaces at only 50% of the regular price and provides a subsidy of $15,000 per vehicle purchase [6]. These subsidies can also be in non-monetary form, such as the granting to shared cars of access to dedicated bus lanes in Norway [29]. Currently, studies focused on exploring subsidy schemes for shared infrastructure and assessing the impact of vehicle purchase subsidies. In looking at how to subsidize infrastructure, scholars have proposed different viewpoints. Some studies suggest that subsidies can be made directly to service providers as monetary income [30–32]. For example, based on the stylized game-theoretic model, Zhang and Dou [31] found that directly subsidizing charging facilities is the most effective subsidy policy for providers that adopt electric car-sharing. Other studies suggest that the government can achieve incentive objectives by collaborating with service providers to construct infrastructure [33, 34]. The typical collaborating mode is the PPP mode, which has advantages such as reducing the financial burden and improving profitability [35].

As for evaluating the efficiency of subsidies, existing studies have approached this from various perspectives. Some studies have found that service providers often rely heavily on subsidy policies, and the removal of subsidies may pose a threat to their survival. This is because of factors such as the travel habits, and accessibility challenges, which have sustained a high level of private car ownership in various countries [36]. Additionally, the car-sharing industry involves substantial capital investment [37]. Some studies have also found that subsidies affect the type of cars adopted by providers [38, 39]. For example, Liu et al. [39], based on a game model, found that, if the government provides a subsidy, there will be more shared cars that are high-end electric vehicles. Otherwise, there will be more low-end vehicles used in car-sharing. In addition, there are studies exploring the potential influence of subsidies on the service quality of providers. For example, based on simulation studies, some scholars have suggested that subsidies to providers can lead to improvements in service quality [40].

#### 2.1.2 Government subsidies to car-sharing users

In recent years, as some countries have begun subsidizing car-sharing users, scholars have started to explore related problems. However, there are currently limited studies on this topic. Some studies have found that subsidies to users indeed increase the acceptance of car-sharing by travelers [41]. Based on data from different countries, studies using structural equation modeling have confirmed this result [24, 42]. Other studies have built on this. For instance, based on data from Shanghai, Zhou et al. [2] used a system dynamics model to simulate the impact of government subsidies on travelers’ preferences. They found that large subsidies were less efficient than smaller ones. This is because larger subsidies may lead the users of public transportation to switch to car-sharing, resulting in subsidy wastage. Other scholars have conducted comparative studies. For example, one study compared the efficiency of government subsidies paid to users and service providers, revealing that subsidizing users yielded better effects [24].

### 2.2 The efficiency of government subsidies

Putting a government subsidies policy in place is important, but the efficiency of the subsidies is an even greater concern [36, 43]. This is because thoughtful subsidies may prove ineffective after implementation, one example being transit subsidies that were offered to different population groups in Stockholm [44]. Meanwhile, an assessment of transport subsidies implementation in Colombia, based on user travel card data for low-income individuals, revealed that the subsidy strategy did effectively increase public transport utilization in that case [45]. In most situations, subsidies can spur demand growth. For example, sales of new electric vehicles in China would “fall off a cliff” if the subsidies were decreased [46], and the government would need to take other actions quickly to prevent this [47]. In addition, when assessing the impact of government subsidies on car-sharing services, it is essential to prioritize feedback from travelers, most of which can be obtained through surveys and empirical studies. Ren et al. [44] conducted a survey among railway passengers to examine the utilization of car-sharing for the first- and last-mile connections with railway stations. Their findings indicated that subsidizing low-income users would be conducive to increasing the adoption of car-sharing as a travel mode. Hu et al. [48] conducted an online survey on new-energy car-sharing vehicles in China, and the results showed that users would be inclined to adopt this emerging travel mode if targeted support and promotional measures were implemented. Furthermore, a survey revealed that car-sharing demand is significantly influenced by users’ preferences in Beijing, China [49], necessitating a comprehensive consideration of diverse user characteristics in government subsidy policies.

### 2.3 Influential factors towards adoption of car-sharing

Travel characteristics, market conditions, and fees are crucial factors influencing travelers’ adoption of car-sharing. As they relate to the travel costs and demand for car-sharing, these factors can potentially affect the efficiency of subsidy policies, altering their reduction of car-sharing travel costs.

Travel characteristics include travel duration, travel mileage, time taken to pick up and return the vehicle, and the weather [28, 50], among others. Existing research finds that car-sharing demand is higher for short-mileage trips, in which car-sharing offers a cost advantage [51–53]. Regarding travel duration, travelers with short-duration trips typically have a higher willingness to use car-sharing. Compared to private cars and other travel modes, car-sharing provides a greater cost advantage for short-duration trips [5, 28]. The time taken to pick up and return the vehicle is a typical feature of car-sharing services. Previous research indicates that it significantly impacts the travel cost of car-sharing [28]. The longer it takes the user to pick up and return the vehicle, the higher is the cost of traveling by car-sharing, which reduces the effectiveness of any subsidy policy in lowering the travel cost. Additionally, weather conditions can impact the demand for car-sharing. For instance, rainy weather can affect road conditions and visibility, thereby diminishing the overall experience and people’s willingness to use car-sharing services [54].

Market conditions refer to the characteristics and situation of a particular market at a particular point in time [55], encompassing factors such as demand elasticity, market potential, and user scale. Market conditions are related to the level of demand for car-sharing and may impact the costs and benefits of subsidy policies. Some studies have found that market conditions impact subsidy efficiency [27]. Demand elasticity reflects the sensitivity of travelers to the cost of traveling by car-sharing. Ren et al. [56] indicated that car-sharing users are generally sensitive to price. This means that car-sharing demand is significantly influenced by elasticity, which can distort subsidy efficiency. The number of car-sharing users also affects travelers’ willingness to adopt car-sharing. Münzel et al. [57] show that the more car-sharing users there are in a region, the more travelers are willing to use car-sharing. Therefore, subsidy policies may be more effective if there are more car-sharing users, as the subsidy will further enhance the benefits of car-sharing. Market potential refers to the number of potential users in the local car-sharing market, which reflects the development potential of car-sharing. Previous research has shown that market potential significantly influences car-sharing policies [58]. For example, car-sharing operators may deploy more stations in areas with high market potential to achieve higher revenues [59]. Therefore, it is essential to consider the impact of market potential on subsidies.

The fees of car-sharing determine the cost of shared travel, thereby affecting the demand for shared travel. Existing research indicates that the fees associated with car-sharing primarily include service fees, pick-up and return costs, and parking fees [51], [60, 61]. Service fees, which are mainly based on travel mileage and travel duration [12], constitute a significant part of the car-sharing costs. Pick-up and return costs reflect the opportunity cost of car-sharing, usually measured in terms of the traveler’s unit time cost [20]. These fees are an important part of the travel cost, and subsidy policies aim to reduce the negative impact of these fees on travelers’ demand for car-sharing.

### 2.4 Review

Previous studies on government subsidies for car-sharing have yielded a wealth of findings. However, they have primarily concentrated on subsidies provided to car-sharing service providers, while overlooking the car-sharing users, relatively speaking. This study specifically focuses on car-sharing users and aims to explore how the government can design subsidy policies to better encourage traveling by car-sharing instead of private cars. Existing studies on subsidizing users have firstly examined the subsidies’ impact on the users’ willingness to utilize car-sharing services, revealing that subsidies do enhance users’ propensity to select car-sharing. Furthermore, scholars have explored differences in the subsidy efficiency in different scenarios, such as under different subsidy amounts and for different recipients.

However, there are still gaps in the previous research that warrant further exploration. The first is the lack of consideration for subsidy costs. When considering subsidy costs, government may choose to withdraw subsidies, making the subsidy policies unsustainable and potentially causing a sudden drop in demand, known as the “cliff effect” [46]. Secondly, there are few studies exploring the impact of different market conditions and travel characteristics on subsidy efficiency. We select market potential, demand elasticity, and number of car-sharing users to reflect market conditions. These three variables influence the travelers’ demand for car-sharing, thereby affecting the costs and benefits of subsidy policies and ultimately affecting their efficiency. And we select travel mileage, travel duration, and time taken to pick up and return the vehicle to reflect the travel characteristics. Travel mileage and travel duration are the most representative travel characteristics because they most directly reflect travelers’ extent of usage and habits concerning car-sharing [8, 62–64]. The time taken to pick up and return the vehicle is a typical feature of car-sharing services. Exploring the influence of these characteristics on subsidies provides a reference for the development of targeted subsidy policies based on different travelers.

Thus, in order to expand on the existing studies, this paper analyzes the impact of subsidy costs on the government and users, delving into the sustainability of subsidy policies. Additionally, it explores, under different subsidy modes, the influence of market conditions and travel characteristics on subsidy efficiency.

## 3. Methodology

### 3.1 Evolutionary game theory

Evolutionary game theory, initially proposed by Smith and Price [65], is founded on the principle of bounded rationality and assumes that a single player in a game represents a population consisting of numerous individuals. The ultimate strategic choice of a given population is determined by the choices made by its individual members. Specifically, individuals within a given population seek to improve their strategy by continuously imitating and learning from the strategies of other individuals within their own population as well as those in other populations. As enough individuals in a certain population change their strategies, the strategic adjustment of the whole population occurs. This dynamic evolutionary process is the essence of the evolutionary game. Evolutionary game theory has been widely applied in various fields of the social sciences [66–68]. Additionally, some studies have applied it to explore problems with car-sharing subsidies [37].

The evolutionary game model is an appropriate choice for the present paper’s analytical framework. This model affords us the opportunity to witness the strategic evolution of players by manipulating variable values. To begin with, we can simulate the behavioral patterns of both the government and users under various subsidy levels to assess the sustainability of subsidy policies. Secondly, by simulating the travel preferences of users under distinct market conditions and travel characteristics, we can explore the differences in the impact of subsidies across diverse scenarios.

Compared to other studies on subsidies, our model differs in several ways. Firstly, it fully considers the unique travel cost of car-sharing, more accurately reflecting the cost differences between car-sharing and private cars, and thereby enhancing the persuasiveness of our results. Specifically, we incorporate duration and mileage-based service fees, as well as the time costs of picking up and returning vehicles, into the travelers’ payoff functions. These costs are unique to traveling by car-sharing. Secondly, our model takes into account the impact of travel mileage on government strategy, enabling us to derive more in-depth conclusions. Specifically, the externalities of car-sharing are mainly reflected in the usage (travel mileage) of car-sharing [12]. The higher is the usage, the stronger are the externalities. Therefore, when quantifying the environmental benefits of subsidies, we consider the impact of travel mileage. This consideration ensures we find that, under mileage-based subsidies, the government’s subsidy costs are offset by environmental benefits as mileage increases, allowing the government to maintain the subsidy strategy for travelers with long-mileage trips and thus encouraging travelers to continue to choose car-sharing.

**Fig 1:**
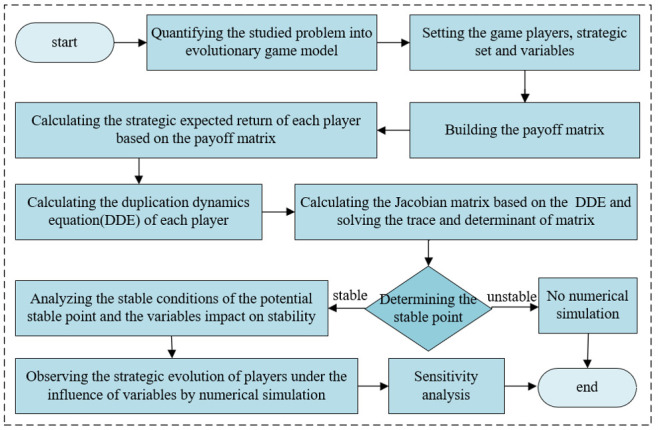
Process of evolutionary game study. Description of the studied process from modeling to numerical simulation.

The process of the evolutionary game study is shown in Fig 1.

### 3.2 Model assumptions

Assumption 1: In the game, there are two players: the travelers and the government, each of whom is boundedly rational and selects strategies to maximize profits.

Assumption 2: The travelers’ strategies are car-sharing and private car. The probabilities of choosing these options are *x* (0<*x*<1) and 1−*x*. The initial probability of selecting car-sharing is *x*_1_. The government’s strategies are subsidy and non-subsidy. The probabilities of choosing these options are *y* (0<*y*<0) and 1−*y*. The initial probability of selecting a subsidy is *y*_1_.

Assumption 3: We make some assumptions for the model to facilitate more comprehensible analysis. This paper does not distinguish between diverse travel experiences or preferences for using car-sharing or a private car, and assumes that each traveler drives individually on a trip and has one and only one private car. Furthermore, this paper does not specify the car-sharing mode because we will adjust the variable representing the time taken to pick up and return the vehicle, which encompasses the variance in car-sharing modes (from the perspective of time costs, the difference between the car-sharing modes mainly comes from the time taken to pick up and return the vehicle). In addition, we assume that the subsidy type is usage-based rather than a lump-sum subsidy, as previous research has shown that usage-based subsidies are more effective than lump-sum subsidies [24].

Assumption 4: The total strategic costs for the traveler of car-sharing and private car use are $\left(P_{m}M+P_{t}T_{1}{+C_{p1}+C}_{t}T_{2})Q(1-\frac{{TC}_{CS}-{TC}_{PC}}{{TC}_{PC}}E_{d}\right)$ and $\left((C_{o}+C_{s}+C_{I})M+C_{p2})Q(1-\frac{{TC}_{CS}-{TC}_{PC}}{{TC}_{PC}}E_{d}\right)$. When travelers select the car-sharing strategy, the strategic costs primarily include the fees of using the car-sharing service, *P*_*m*_*M*+*P*_*t*_*T*_1_, parking costs *C*_*p*1_, and the time costs incurred in picking up and returning the vehicle, *C*_2_*T*_2_ [5], [69]. *M* represents the travel mileage, *T*_1_ is the travel duration, and *T*_2_ is the time spent picking up and returning the vehicle. *P*_*m*_ and *P*_*t*_ refer to the unit mileage and time fees of using car-sharing services. *C*_*p*1_ denotes the parking fees during travel. *C*_*t*_ represents the opportunity cost per unit of time. When travelers select the private car strategy, the strategic costs are represented by the travel costs of the private car, which mainly include parking costs *C*_*p*2_, the maintenance costs of private cars *C*_*s*_*M*, the fuel costs of private cars *C*_*o*_*M*, and the insurance costs of private cars *C*_*I*_*M* [28, 70]. *C*_*p*2_ stands for the parking fees when traveling or at home. *C*_*s*_ represents the maintenance fees, such as cleaning, per unit of mileage. *C*_*o*_ stands for the fuel costs per unit of mileage for private cars. *C*_*I*_ refers to the insurance fees for private cars per unit of mileage. The insurance costs depend on the travel mileage, because long-mileage travelers tend to pay higher insurance premiums due to the increased likelihood of accidents associated with greater mileage. They therefore have a greater demand for car insurance, and insurance companies adjust their rates based on drivers’ records [71–73]. For example, Progressive Insurance (an insurance company) has been charging insurance fees based on travel mileage since 1994 [71]. In addition, we assume the number of travelers to be *Q*, which reflects travelers’ potential demand for car-sharing. When travelers are deciding whether to change from traveling by private car to car-sharing, if the travel cost of car-sharing is higher, the demand for car-sharing will be lower, and some travelers will continue to use private cars. In other words, travelers’ demand for car-sharing is elastic. Therefore, travelers’ demand for car-sharing is assumed to be $Q(1-\frac{{TC}_{CS}-{TC}_{PC}}{{TC}_{PC}}E_{d})$, where *E*_*d*_ represents the absolute value of the price elasticity of demand for car-sharing, *TC*_*CS*_ represents the travel cost of car-sharing, *P*_*m*_*M*+*P*_*t*_*T*_1_+*C*_*p*1_+*C*_*t*_*T*_2_, and *TC*_*PC*_ represents the travel cost of private cars, (*C*_*o*_+*C*_*s*_+*C*_*I*_)*M*+*C*_*p*2_. To ensure that the demand is greater than 0, we assume $\frac{{TC}_{CS}-{TC}_{PC}}{{TC}_{PC}}E_{d}<1$.

Assumption 5: The government’s strategic payoffs from the subsidy and non-subsidy strategies are $\left(BM-S)Q(1-\frac{{TC}_{CS}-{TC}_{PC}-S}{{TC}_{PC}}E_{d}\right)$ and $\frac{BMQ(1-\frac{{TC}_{CS}-{TC}_{PC}}{{TC}_{PC}}E_{d})}{\delta }$ if traveler selects the car-sharing strategy. When the government selects the subsidy strategy, it will grant subsidy *S* for each trip (fixed subsidy) to travelers selecting car-sharing strategy. In turn, the government reaps the environmental benefit *BM* from users traveling by car-sharing, where *B* is the monetary cost of gas emissions saved per unit of mileage when utilizing a shared car instead of a private car. The overall strategic payoff of the subsidy strategy amounts to $\left(BM-S)Q(1-\frac{{TC}_{CS}-{TC}_{PC}-S}{{TC}_{PC}}E_{d}\right)$. When the government selects the non-subsidy strategy, it refrains from doling out subsidies but still reaps limited environmental benefit $\frac{BMQ(1-\frac{{TC}_{CS}-{TC}_{PC}}{{TC}_{PC}}E_{d})}{\delta }$ [74], where *δ* is a coefficient, with $0<\frac{1}{\delta }<1$. When the government provides subsidies, users are more likely to adopt car-sharing earlier. In the absence of government subsidies, users may take a considerable time before being willing to adopt car-sharing. As a result, the government’s environmental benefits are lower when they select the non-subsidy strategy.

We use the traveler’s initial probability of selecting car-sharing *x*_1_, the number of travelers *Q*, and the demand elasticity of car-sharing *E*_*d*_ to represent the car-sharing market conditions. The traveler’s initial probability of selecting car-sharing reflects the scale of existing users of car-sharing, and the number of travelers indicates the potential of the car-sharing market. Additionally, we employ the travel mileage *M*, travel duration *T*_1_, and time spent picking up and returning the vehicle *T*_2_ to reflect the travel characteristics.

All variables are listed in Table 1.

**Table 1:** Model variables.

Variable	Meaning	Variable	Meaning
*Q*	Number of travelers	*C*_*s*_	Maintenance cost of private cars
*M*	Travel mileage	*C*_*I*_	Insurance fees of private cars
*T*_1_	Travel duration	*C*_*o*_	Fuel cost of private cars
*P*_*m*_	Rental fee(mileage)	*B*	Environmental benefits from adopting car-sharing
*P*_*t*_	Rental fee(time)	*S*	subsidy to travelers
*C*_*t*_	Opportunity cost of picking up and returning vehicle	*δ*	Coefficient
*T*_2_	Time spent picking up and returning vehicle	*E*_*d*_	Demand elasticity
*C*_*p*1_	Parking fee of car-sharing	*x*_1_	Traveler’s initial probability of selecting car-sharing
*C*_*p*2_	Parking fee of private cars	*y*_1_	Government’s initial probability of providing a subsidy

**Fig 2:**
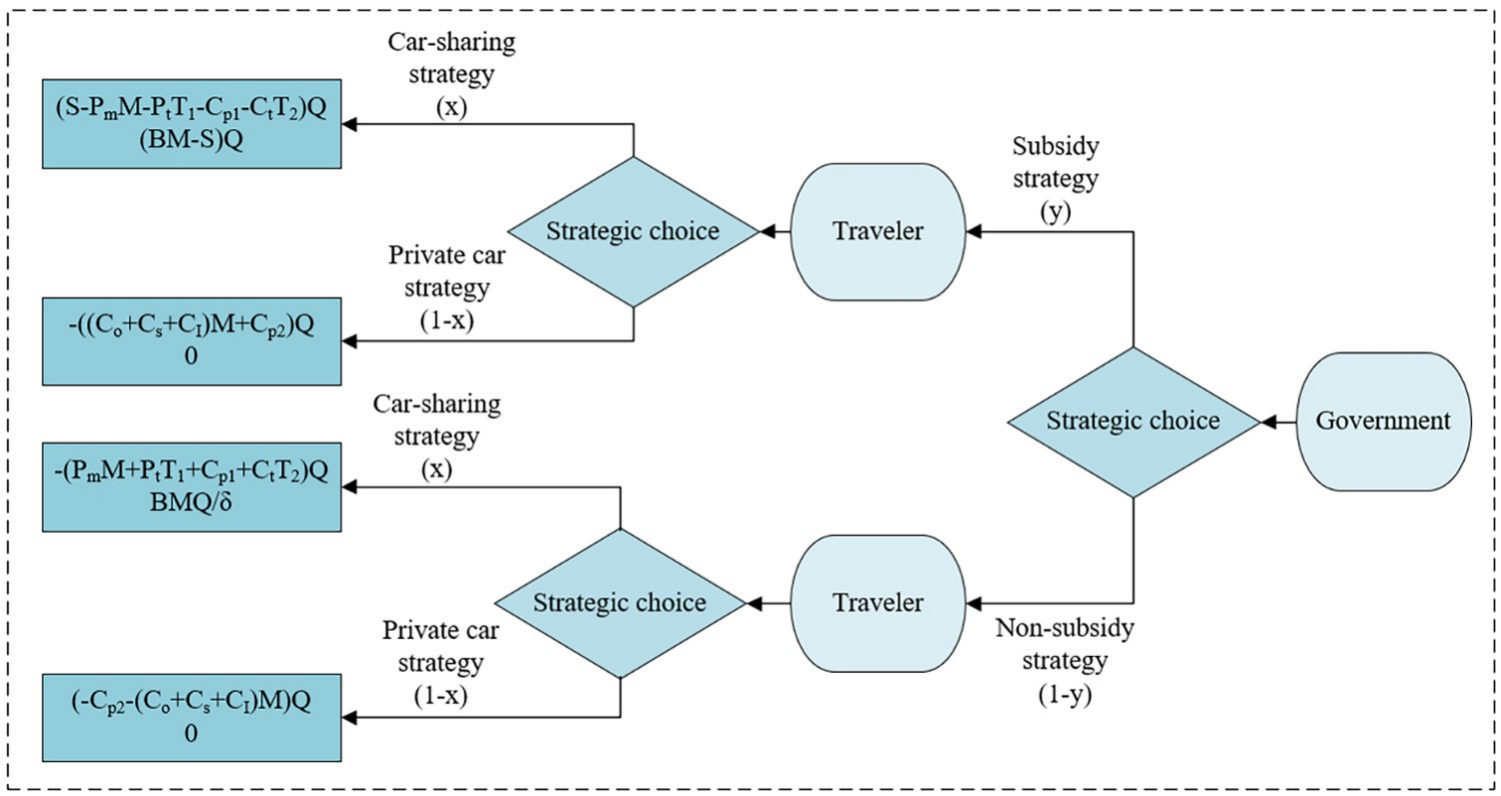
Tree diagram of game. Description of players’ strategy combinations and corresponding payoff.

Based on the above assumptions, we draw a tree diagram to reflect the game between the government and the travelers and their strategic benefits, as shown in Fig 2.

### 3.3 Payoff matrix and replication dynamics equation

Based on Section 3.2, the payoff matrix of the game can be given as in Table 2.

**Table 2:** Payoff matrix.

	Government		
		Subsidy *y* = 1	Non-subsidy *y* = 0
Traveler	Car-sharing *x* = 1	Traveler: $\left(S-P_{m}M-P_{t}T_{1}{-C_{p1}-C}_{t}T_{2})Q(1-\frac{{TC}_{CS}-{TC}_{PC}-S}{{TC}_{PC}}E_{d}\right)$ Government: $\left(BM-S)Q(1-\frac{{TC}_{CS}-{TC}_{PC}-S}{{TC}_{PC}}E_{d}\right)$	Traveler: $\left(-P_{m}M-P_{t}T_{1}-C_{p1}-C_{t}T_{2}\right)$ $Q(1-\frac{{TC}_{CS}-{TC}_{PC}}{{TC}_{PC}}E_{d})$ Government: $\frac{BMQ(1-\frac{{TC}_{CS}-{TC}_{PC}}{{TC}_{PC}}E_{d})}{\delta }$
	Private car *x* = 0	Traveler: $-((C_{o}+C_{s}+C_{I})M+C_{p2})Q(1-\frac{{TC}_{CS}-{TC}_{PC}-S}{{TC}_{PC}}E_{d})$ Government: 0	Traveler: $-((C_{o}+C_{s}+C_{I})M+C_{p2})Q(1-\frac{{TC}_{CS}-{TC}_{PC}}{{TC}_{PC}}E_{d})$ Government: 0

According to assumptions 1 and 2, the game has two players: the traveler and the government. The traveler’s and the government’s strategies are car-sharing and private car, subsidy and non-subsidy, so there are four strategic combinations: (car-sharing, subsidy), (car-sharing, non-subsidy), (private car, subsidy), and (private car, non-subsidy). According to assumption 4, when the government chooses the subsidy strategy, the payoff is $\left(BM-S)Q(1-\frac{{TC}_{CS}-{TC}_{PC}-S}{{TC}_{PC}}E_{d}\right)$, and it will provide the subsidy *S* to every traveler choosing car-sharing. According to assumption 3, the traveler’s cost of choosing car-sharing is $\left(P_{m}M+P_{t}T_{1}{+C_{p1}+C}_{t}T_{2})Q(1-\frac{{TC}_{CS}-{TC}_{PC}}{{TC}_{PC}}E_{d}\right)$. Therefore, when the strategy combination is (car-sharing, subsidy), the traveler’s payoff is $\left(S-P_{m}M-P_{t}T_{1}{-C_{p1}-C}_{t}T_{2})Q(1-\frac{{TC}_{CS}-{TC}_{PC}-S}{{TC}_{PC}}E_{d}\right)$ and the government’s payoff is $\left(BM-S)Q(1-\frac{{TC}_{CS}-{TC}_{PC}-S}{{TC}_{PC}}E_{d}\right)$. When the strategy combination is (car sharing, non-subsidy), the government will not provide subsidies. According to assumptions 3 and 4, the payoffs of the travelers and the government are then $\left(-P_{m}M-P_{t}T_{1}{-C_{p1}-C}_{t}T_{2})Q(1-\frac{{TC}_{CS}-{TC}_{PC}}{{TC}_{PC}}E_{d}\right)$ and $\frac{BMQ(1-\frac{{TC}_{CS}-{TC}_{PC}}{{TC}_{PC}}E_{d})}{\delta }$ respectively. When the strategy combination is (private car, subsidy) or (private car, non-subsidy), the government will not obtain an environment benefit or provide a subsidy, and so its payoff is 0. The traveler’s payoff is the travel cost of the private car.

Then, the traveler’ s expected return function from selecting car-sharing *U*_1_, from selecting the private car *U*_2_, and the average return function $\bar{U}$ can be written as:

$$U_{1}=y(S-P_{m}M-P_{t}T_{1}{-C_{p1}-C}_{t}T_{2})Q_{1}+(1-y)(-P_{m}M-P_{t}T_{1}{-C_{p1}-C}_{t}T_{2})Q_{2}$$ (1)

$$U_{2}=-y((C_{o}+C_{s}+C_{I})M+C_{p2})Q_{1}+(1-y)(-(C_{o}+C_{s}+C_{I})M-C_{p2})Q_{2}$$ (2)

$$\bar{U}=xU_{1}+(1-x)U_{2}=y(x(S-P_{m}M-P_{t}T_{1}-C_{p1}-C_{t}T_{2})Q_{1}-(1-x)((C_{o}+C_{s}+C_{I})M+C_{p2})Q_{1})+(1-y)(x(-P_{m}M-P_{t}T_{1}-C_{p1}-C_{t}T_{2})Q_{2}+(1-x)(-(C_{o}+C_{s}+C_{I})M-C_{p2})Q_{2})$$ (3)

where *Q*_1_ represents $Q(1-\frac{{TC}_{CS}-{TC}_{PC}-S}{{TC}_{PC}}E_{d})$ and *Q*_2_ represents $Q(1-\frac{{TC}_{CS}-{TC}_{PC}}{{TC}_{PC}}E_{d})$.

The expected return function when the government selects the subsidy *V*_1_, when it selects the non-subsidy strategy *V*_2_, and the average return function $\bar{V}$ can be written as:

$$V_{1}=x(BM-S)Q_{1}+(1-x)*0=x(BM-S)Q_{1}$$ (4)

$$V_{2}=x\frac{BMQ_{2}}{\delta }+(1-x)*0=x\frac{BMQ_{2}}{\delta }$$ (5)

$$\bar{V}=yV_{1}+(1-y)V_{2}=x\left(y(BM-S)Q_{1}+(1-y)\frac{BMQ_{2}}{\delta }\right)$$ (6)

Next, we give the replication dynamics equations for both the traveler and the government. Replication dynamics equations are derived based on the Malthusian equation [75]. The expression of Malthusian equation is as follows:

$$\frac{dn_{i}}{dt}=n_{i}*r_{i}$$ (7)

In Eq (7), *n* represents the population size of some kind of creature, while *n*_*i*_ represents the number of individuals of type *i* (where *i* = 1,2,…,*k*, and $n=\sum_{i=1}^{k}n_{i}$) within this population. *r*_*i*_ represents the growth rate of individuals’ number of type *i*. Eq (7) reflects the change in the individuals’ number of type *i* with respect to time.

Then by following a change of coordinates,

$$q_{i}=\frac{n_{i}}{n}$$ (8)

*q*_*i*_ represents the proportion of individuals of type *i* within the population. Taking the derivative of *q*_*i*_ with respect to, there is:

$$\frac{dq_{i}}{dt}=\frac{d}{dt}(\frac{n_{i}}{n})=\frac{\frac{dn_{i}}{dt}*n-n_{i}*\frac{dn}{dt}}{n^{2}}$$ (9)

From $\frac{dn_{i}}{dt}=n_{i}*r_{i}$ and $\frac{dn}{dt}=\sum_{i=1}^{k}\frac{dn_{i}}{dt}$, there is:

$$\frac{dq_{i}}{dt}=\frac{n_{i}*r_{i}*n-n_{i}*\sum_{i=1}^{k}n_{i}*r_{i}}{n^{2}}=\frac{n_{i}*r_{i}}{n}-\frac{n_{i}*\sum_{i=1}^{k}n_{i}*r_{i}}{n^{2}}$$ (10)

Where, $\frac{\sum_{i=1}^{k}n_{i}*r_{i}}{n}$ represents the average growth rate of the creature, denoted as $\bar{r}$.

Finally, there is:

$$\frac{dq_{i}}{dt}=q_{i}*r_{i}-q_{i}*\bar{r}=q_{i}*(r_{i}-\bar{r})$$ (11)

Based on Eq (11), we write the replication dynamics equation of the traveler as:

$$dx(t)={x(t)*(U}_{1}-\bar{U})={x(t)*(U}_{1}-x{*U}_{1}-(1-x){*U}_{2})=x(t)*(1-x(t))*(U_{1}-U_{2})=x(t)*(1-x(t))*(y{*Q}_{1}*(S-P_{m}*M-P_{t}{*T}_{1}{-C_{p1}-C}_{t}{*T}_{2}+(C_{o}+C_{s}+C_{I})*M+C_{p2})-(1-y){*Q}_{2}*(P_{m}*M+P_{t}{*T}_{1}+C_{p1}+C_{t}{*T}_{2}-(C_{o}+C_{s}+C_{I})*M-C_{p2}))d(t)$$ (12)

The replication dynamics equation of the government can be written as:

$$dy(t)={y(t)*(V}_{1}-\bar{V})={y(t)*(V}_{1}-y*V_{1}-(1-y)*V_{2})=y(t)*(1-y(t))*(V_{1}-V_{2})=y(t)*(1-y(t))*(x*(B*M*(Q_{1}-\frac{Q_{2}}{\delta })-S{*Q}_{1}))d(t)$$ (13)

### 3.4. Conditions of the strategic equilibrium and stability

According to the stability theory of differential equations, in order for the strategic combination of the traveler and the government to be an evolutionary stable strategy (ESS), the following conditions must be met:

$$dx(t)=0,\;\frac{\partial dx(t)}{\partial x}<0,\;dy(t)=0,\;\frac{\partial dy(t)}{\partial y}<0$$

Where,

$$\frac{\partial dx(t)}{\partial x}=(1-2*x(t))*(y{*Q}_{1}*(S-P_{m}*M-P_{t}{*T}_{1}{-C_{p1}-C}_{t}{*T}_{2}+(C_{o}+C_{s}+C_{I})*M+C_{p2})-(1-y){*Q}_{2}*(P_{m}*M+P_{t}{*T}_{1}{+C_{p1}+C}_{t}{*T}_{2}-(C_{o}+C_{s}+C_{I})*M-C_{p2}))d(t)$$ (14)

$$\frac{\partial dy(t)}{\partial y}=(1-2*y(t))*(x*(B*M*(Q_{1}-\frac{Q_{2}}{\delta })-S{*Q}_{1}))d(t)$$ (15)

By making Eqs (12) and (13) equal to 0, it can be deduced that *dx*(*t*) = 0 when *x* = 0 or *x* = 1, and *dy*(*t*) = 0 when *y* = 0 or *y* = 1. Therefore, the corresponding equilibrium points are *E*_1_(1,1), *E*_2_(1,0), *E*_3_(0,1), *E*_4_(0,0). *E*_1_(1,1) means the traveler selects car-sharing and the government selects a subsidy. The meanings of the other points can be deduced in the same way. A non-end point can also be the equilibrium point, but in evolutionary games, a non-end point equilibrium point is unstable [76], so we do not consider this.

Next, we will discuss the stable conditions for the four equilibrium points. When the equilibrium point is an ESS, its corresponding replicated dynamic equations (Eqs (14) and (15)) must satisfy $\frac{\partial dx(t)}{\partial x}<0$ and $\frac{\partial dy(t)}{\partial y}<0$.

In order for *E*_1_(1,1) to be an ESS, which means the traveler’s strategy stabilizes at car-sharing and the government’s strategy stabilizes at providing a subsidy, the following two conditions need to be met:

$${\frac{\partial dx(t)}{\partial x}}_{x=1}<0,{\frac{\partial dy(t)}{\partial y}}_{y=1}<0$$

That is,

$$y>y^{*}=\frac{Q_{2}}{Q_{1}*(1+\frac{S}{(C_{o}+C_{s}+C_{I})*M+C_{p2}-C_{p1}-P_{m}*M-P_{t}{*T}_{1}{-C}_{t}*T_{2}})-Q_{2}}$$ (16)

$$x>0\;and\;BM\left(Q_{1}-\frac{Q_{2}}{\delta }\right){>SQ}_{1}$$ (17)

Inequality (16) indicates that, when the government’s probability of choosing the subsidy strategy satisfies *y*>y*, the traveler chooses the car-sharing strategy. Based on inequality (16), we find that, as the subsidy *S* increases, and *C*_*p*1_+*P*_*m*_*M*+*P*_*t*_*T*_1_+*C*_*t*_*T*_2_, the travel cost of car-sharing, decreases, the value of $y^{*}=\frac{Q_{2}}{Q_{1}*(1+\frac{S}{(C_{o}+C_{s}+C_{I})*M+C_{p2}-C_{p1}-P_{m}*M-P_{t}{*T}_{1}{-C}_{t}{*T}_{2}})-Q_{2}}$ decreases, which means inequality (17) is easier to establish and the traveler is more likely to choose the car-sharing strategy. Otherwise, the traveler is more likely to choose a private car.

**Fig 3:**
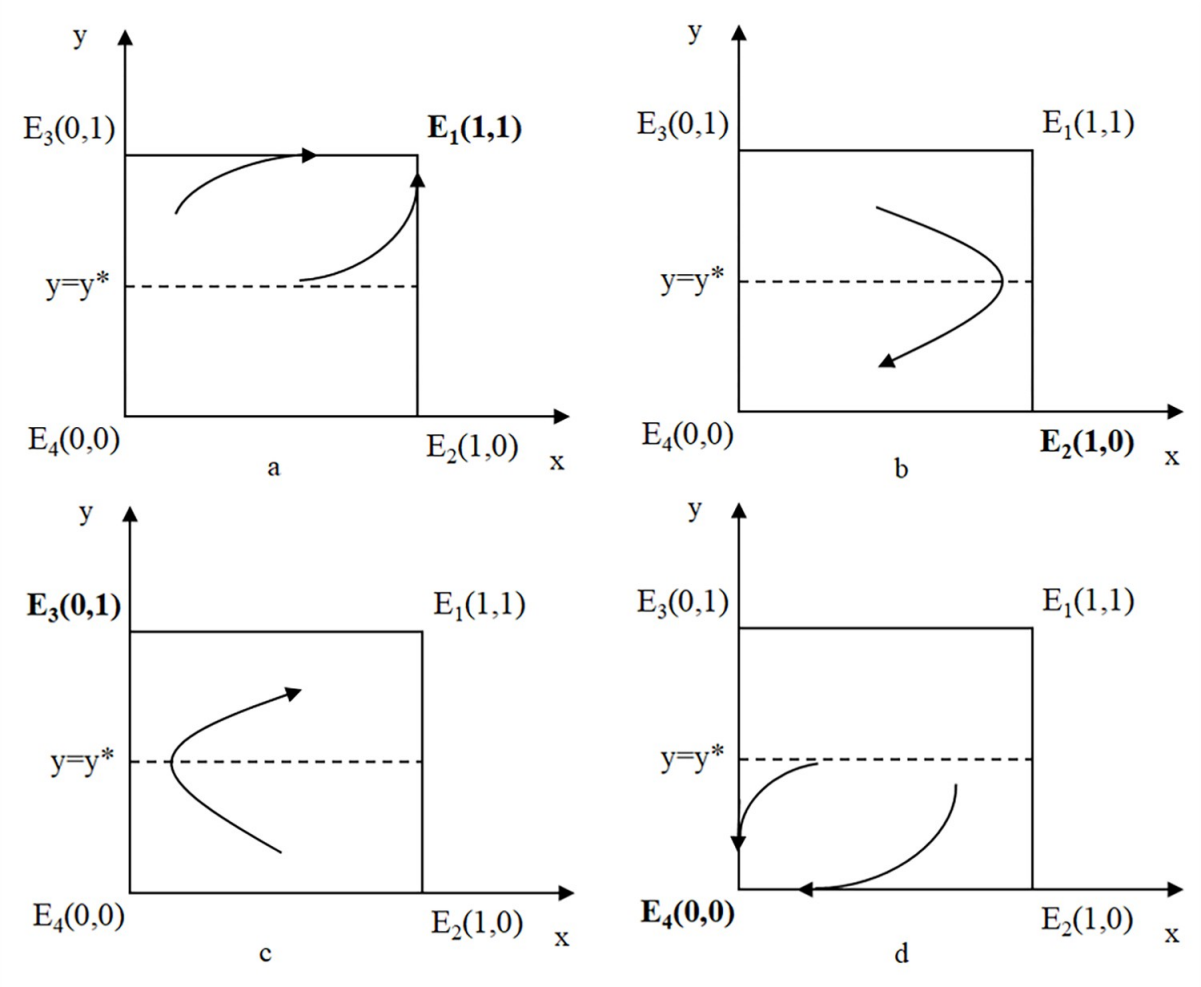
Phase diagram of four potential stable points. (a) Description of strategy combination evolving towards and stabilizing at (1,1). (b) Description of strategy combination evolving towards (1,0). (c) Description of strategy combination evolving towards (0,1). (d) Description of strategy combination evolving towards and stabilizing at (0,0).

Inequality (17) indicates that, when the traveler’s probability of choosing the car-sharing strategy is non-zero, and the environmental benefit $BM(Q_{1}-\frac{Q_{2}}{\delta })$ is higher than the subsidy cost *SQ*_1_, the government will choose the subsidy strategy. Fig 3(A) shows the strategic evolution of the traveler and government towards *E*_1_(1,1) when inequalities (16) and (17) hold. In Fig 3(A), there are two evolutionary paths that can be taken for *E*_1_(1,1) to become an ESS. One is that the government’s subsidy strategy first evolves to a stable strategy, and then the car-sharing strategy of the traveler evolves to a stable strategy. Specifically, when the environmental benefits exceed the subsidy costs, the government’s willingness to choose the subsidy strategy increases, gradually evolves to and then stabilizes at the subsidy strategy. With government subsidies, the travel cost of car-sharing decreases, thereby increasing travelers’ willingness to use shared travel (car-sharing), and the traveler ultimately evolves to and then stabilizes at the car-sharing strategy. The other path is that, when the service fees and other fees of car-sharing are low, compared to private cars, travelers using car-sharing have lower travel costs, resulting in a stronger willingness to use shared travel. The traveler’s car-sharing strategy then ultimately becomes the stable strategy. As the proportion of travelers choosing the car-sharing strategy increases, the more traveling by private cars is replaced by shared travel, thereby resulting in greater environmental benefits. Thus, the government becomes more willing to choose the subsidy strategy, which ultimately becomes the stable strategy.

In order for *E*_2_(1,0) to become an ESS, which means the traveler’s strategy stabilizes at car-sharing and the government’s strategy stabilizes at non-subsidy, the following two conditions need to be met:

$${\frac{\partial dx(t)}{\partial x}}_{x=1}<0,{\frac{\partial dy(t)}{\partial y}}_{y=0}<0$$

That is,

$$y>y^{*}=\frac{Q_{2}}{Q_{1}*(1+\frac{S}{(C_{o}+C_{s}+C_{I})*M+C_{p2}-C_{p1}-P_{m}*M-P_{t}{*T}_{1}{-C}_{t}*T_{2}})-Q_{2}}$$ (18)

$$x>0\;and\;BM\left(Q_{1}-\frac{Q_{2}}{\delta }\right){<SQ}_{1}$$ (19)

However, the phase diagram Fig 3(B) shows that *E*_2_(1,0) cannot become an ESS. Specifically, when *y*>y*, *x*>0, and $BM(Q_{1}-\frac{Q_{2}}{\delta }){<SQ}_{1}$ are satisfied, the evolutionary path of the strategy combination will move to the lower right, but as *y*>y* no longer holds, the evolutionary path begins to move to the lower left, as shown by the arrow in the Fig 3(B). Therefore, *E*_2_(1,0) cannot become an ESS. The reason is that, at the beginning of the game, the government has a higher willingness to subsidize traveling by car-sharing, and the traveler gradually evolves towards the car-sharing strategy. However, due to the high cost of the subsidy, the willingness of the government to provide it starts to decline, the government’s non-subsidy strategy eventually becomes the ESS, and then the traveler’s willingness to use shared travel also decreases and the private car strategy becomes the ESS.

In order for *E*_3_(0,1) to become an ESS, which means the traveler’s strategy stabilizes at the private car and the government’s strategy stabilizes at the subsidy, the following two conditions need to be met:

$${\frac{\partial dx(t)}{\partial x}}_{x=0}<0,{\frac{\partial dy(t)}{\partial y}}_{y=1}<0$$

That is,

$$y<y^{*}=\frac{Q_{2}}{Q_{1}*(1+\frac{S}{(C_{o}+C_{s}+C_{I})*M+C_{p2}-C_{p1}-P_{m}*M-P_{t}{*T}_{1}{-C}_{t}*T_{2}})-Q_{2}}$$ (20)

$$x>0\;and\;BM\left(Q_{1}-\frac{Q_{2}}{\delta }\right){>SQ}_{1}$$ (21)

The phase diagram Fig 3(C) shows that *E*_3_(0,1) cannot become an ESS. Specifically, when *y*<y*, *x*>0, and $BM(Q_{1}-\frac{Q_{2}}{\delta }){>SQ}_{1}$ are satisfied, the evolutionary path will move to the upper right. However, as *y*<y* no longer holds, the evolutionary path moves to the upper left, as shown by the arrow in the Fig 3(C). Therefore, *E*_3_(0,1) cannot become an ESS. Specifically, as the environmental benefits are higher, the government has a higher willingness to subsidize and gradually evolves towards the subsidy strategy. The subsidy reduces the cost of shared travel, thereby increasing the willingness of travelers to use shared travel, rather than evolving towards the private car strategy.

In order for *E*_4_(0,0) to become an ESS, which means the traveler’s strategy stabilizes at the private car and the government’s strategy stabilizes at non-subsidy, the following two conditions need to be met:

$${\frac{\partial dx(t)}{\partial x}}_{x=0}<0,\;{\frac{\partial dy(t)}{\partial y}}_{y=0}<0$$

That is.


$$y<y^{*}=\frac{Q_{2}}{Q_{1}*(1+\frac{S}{(C_{o}+C_{s}+C_{I})*M+C_{p2}-C_{p1}-P_{m}*M-P_{t}{*T}_{1}{-C}_{t}*T_{2}})-Q_{2}}$$ (22)

$$x>0\;and\;BM\left(Q_{1}-\frac{Q_{2}}{\delta }\right){<SQ}_{1}$$ (23)

Inequalities (22) and (23) indicate that, when the shared travel cost is high and the subsidy cost is higher than the environmental benefits, the ESS of the game will be *E*_4_(0,0). In the phase diagram shown in Fig 3(D), it can be seen that there are two paths that can be followed for *E*_4_(0,0) to become the ESS. One is that the government’s non-subsidy strategy first becomes the stable strategy, and then the private car strategy of the traveler becomes the stable strategy. Specifically, when the cost of the subsidy is high, the government’s subsidy strategy has a lower payoff then gradually evolves into an unstable strategy. Without the subsidy, the cost of shared travel increases rapidly, and the private car strategy becomes the stable strategy. The other path is the opposite. When the cost of shared travel is still high under a subsidy policy, the willingness of a traveler to travel by car-sharing rapidly decreases and evolves towards the private car strategy. Then, the government evolves to and stabilizes at the non-subsidy strategy, because it cannot obtain the environmental benefits from the subsidy strategy.

The above analysis demonstrates that, firstly, in the game between the travelers and the government, only *E*_1_(1,1) and *E*_4_(0,0) can become ESSs, indicating the importance of government subsidies. Without government subsidies, travelers cannot evolve to a car-sharing strategy. Secondly, the strategic choice of the travelers depends on the travel cost of car-sharing, while the government’s strategic choice depends on the balance between the environmental benefits and subsidy costs. Next, we conduct a further exploration using numerical simulations, providing a reference for governments to use to better design subsidy policies and encourage shared travel.

## 4. Numerical simulation

We used MATLAB (version 2021) to simulate and analyze the evolutionary game results. The relevant data and code are shown in S1 Appendix. The data for the numerical simulations comes from Beijing. In 2022, the number of private cars in Beijing was 4.93 million, so we set *Q* to this value (assuming each traveler has one car). In 2022, in Beijing, the average mileage and duration of trips by private car were 13.89 km and 36.71 min, respectively. Therefore, the initial values of *M* and *T*_1_ are set to these values, respectively. Based on GoFun (China’s most popular car-sharing platform), car-sharing rental fees consist of two parts: a time fee of 0.1 CNY per minute, and a mileage fee of 1.5 CNY per kilometer. Therefore, *P*_*t*_ and *P*_*m*_ are set to these values. As mentioned in paper of Hu et al. [5], the time cost of picking up and returning a vehicle, *C*_*t*_, can be calculated as the per capita per minute income of Beijing citizens in 2022, which gives us 0.67 CNY per minute. The time spent picking up and returning a vehicle, *T*_2_, is not precisely known. We give it the initial value of 10 min based on survey research [24]. The value of *C*_*p*1_, the temporary parking fees when traveling by car-sharing, are set to 1.25 CNY, based on data from the Beijing Transportation Development Research Institute. The values of *C*_*p*2_, *C*_*s*_ and *C*_*I*_ are set to 3.19 CNY, 0.26 CNY, and 0.47 CNY, also obtained from that source. The fuel cost of private cars, *C*_*o*_, is set to the average fuel cost per kilometer driven. In 2022, the average fuel consumption of private cars in Beijing was 0.05 liters per kilometer, and the fuel price in Beijing was 7.58 CNY per liter on December 31, 2022, so *C*_*o*_ is set to 0.38 CNY per kilometer. The average reduction in CO_2_ emissions from the adoption of car-sharing is 114.9g per kilometer, and the average carbon price in China in 2022 was 0.03 CNY per gram, giving a value for the environmental benefits *B* of 3.4 CNY. The values of the subsidy *S*, demand elasticity *E*_*d*_, and parameter *δ* are uncertain, but we assign them the initial values of 15 CNY, 1.1, and 5. In addition, we let the travelers’ initial probability of selecting car-sharing *x*_1_ = 0.5 and the government’s initial probability of selecting the subsidy strategy *y*_1_ = 0.5. The values of all the variables are shown in Table 3.

**Table 3:** Values of all variables.

Variable	Meaning	Value	Unit	Real data
*M*	Travel mileage	13.89	Km	Yes
*T*_1_	Travel duration	36.71	Min	Yes
*P*_m_	Rental fee(mileage)	1.5	CNY/km	Yes
*P*_*t*_	Rental fee(time)	0.1	CNY/min	Yes
*C*_*t*_	Opportunity cost of picking up and returning	0.67	CNY/min	Yes
*T*_2_	Time spent picking up and returning	10	Min	Yes
*C*_*p*1_	Parking cost of car-sharing	1.25	CNY /one trip	Yes
*C*_*p*2_	Parking cost of private cars	3.19	CNY/one trip	Yes
*C*_*s*_	Maintenance cost of private cars	0.26	CNY/km	Yes
*C*_*I*_	Insurance cost of private cars	0.47	CNY/km	Yes
*C*_*o*_	Fuel cost of private cars	0.38	CNY/km	Yes
*B*	Environmental benefits of adopting car-sharing	3.4	CNY/km	Yes
*Q*	Number of travelers	4930000	People	Yes
*S*	Subsidy to travelers	15	CNY/one trip	Simulated
*δ*	Coefficient	5	-	Simulated
*E*_*d*_	Demand elasticity for car-sharing	1.1	-	Simulated
*x*_1_	Traveler’s initial probability of selecting car-sharing	0.5	-	Simulated
*y*_1_	Government’s initial probability of selecting subsidy	0.5	-	Simulated

### 4.1 Strategy evolution of players under the subsidy policies

**Fig 4:**
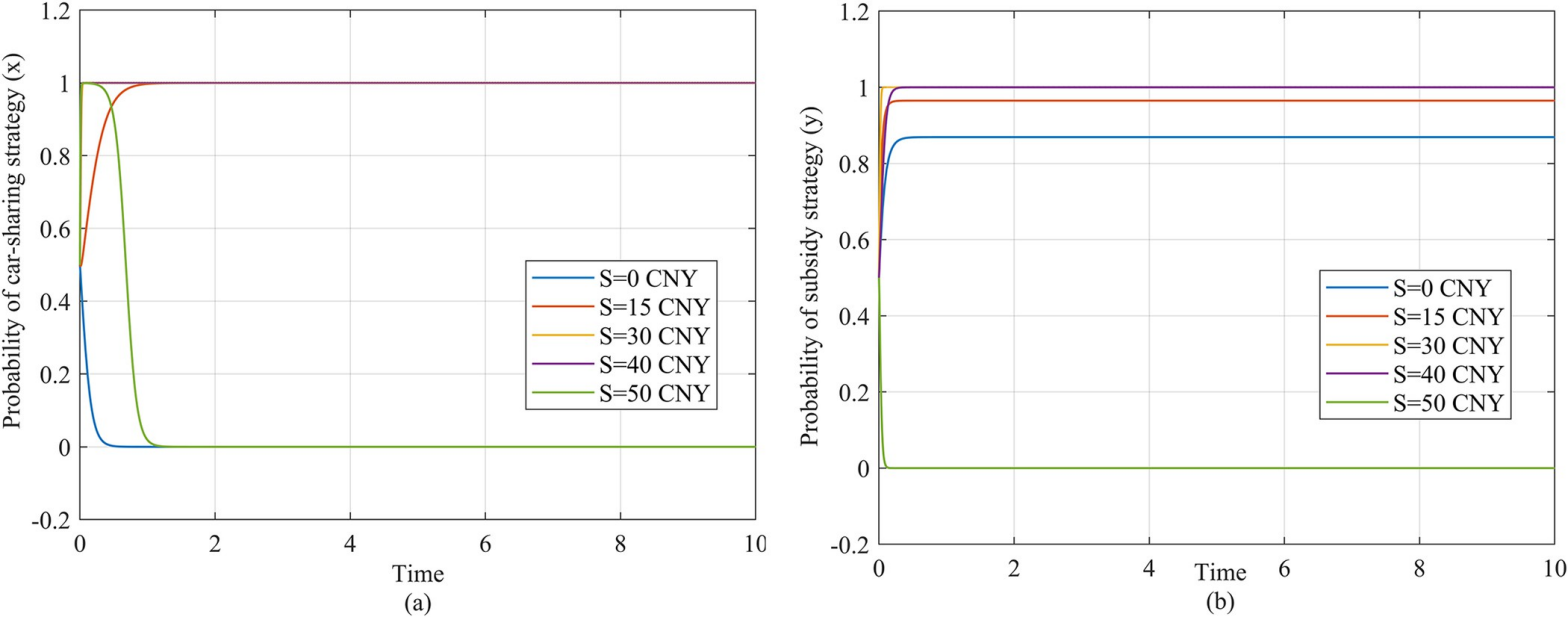
Strategy evolution of players under different subsidy values. (a) Description of the travelers’ strategy evolution as the subsidy value changes. (b) Description of the government’s strategy evolution as the subsidy value changes.

Firstly, we simulate the strategic evolution of both the travelers and the government in response to different values of the subsidy, as illustrated in Fig 4. The horizontal axis represents time, while the vertical axis represents the probability that the traveler selects car-sharing (*x*) or the government selects subsidy (*y*). The function curves reflect the values of *x* or *y* changing over time.

Fig 4(A) reflects the dynamic evolution of the traveler’s strategy. Specifically, when the subsidy *S* is 0 CNY per trip, as shown by the blue curve in Fig 4(A), the traveler will tend to maintain the private car strategy (*x* = 0). When *S* increases to 15 CNY per trip, 30 CNY per trip and 40 CNY per trip as shown by red, yellow and purple curves, the traveler’s strategy quickly evolves to car-sharing (*x* = 1). However, as the subsidy continues to increase, the traveler evolves back to a private car strategy again, as shown by the green curves.

Fig 4(B) illustrates the dynamic evolution of the government’s strategy. When the subsidy *S* is 0, 15, 30 and 40 CNY per trip, as shown by the blue, red, yellow and purple curves in Fig 4(B), the government’s probability of selecting the subsidy strategy (*y*) gradually evolves to *y* = 1, but when *S* is 50 CNY per trip, as shown by the green curves, the government’s probability of selecting the subsidy strategy gradually evolves to *y* = 0.

The simulation shown in Fig 4(A) and 4(B) indicate that the travelers’ and government’s willingness to choose the car-sharing and subsidy strategies will first increase and then decrease with the increase of the subsidy. When the subsidy amount is low, as it increases, the cost of shared travel decreases. The condition for choosing the car-sharing strategy, $y>\frac{Q_{2}}{Q_{1}*(1+\frac{S}{(C_{o}+C_{s}+C_{I})*M+C_{p2}-C_{p1}-P_{m}*M-P_{t}{*T}_{1}{-C}_{t}*T_{2}})-Q_{2}}$, is more likely to be met and the travelers are more likely to choose the car-sharing strategy. As a larger proportion of travelers become willing to choose the car-sharing strategy, the government obtains greater environmental benefits and the condition for choosing the subsidy strategy, $BM(Q_{1}-\frac{Q_{2}}{\delta }){>SQ}_{1}$, is more easily met. Therefore, the government is more willing to choose the subsidy strategy. However, when the subsidy amount is high, the government’s subsidy cost increases rapidly, resulting in $BM(Q_{1}-\frac{Q_{2}}{\delta }){<SQ}_{1}$, which means the government’s strategy evolves to not providing a subsidy. Without the subsidy, the cost of shared travel increases rapidly, and the travelers’ strategy stabilizes to using a private car.

### 4.2 The impact of different factors on the subsidy efficiency

**Fig 5:**
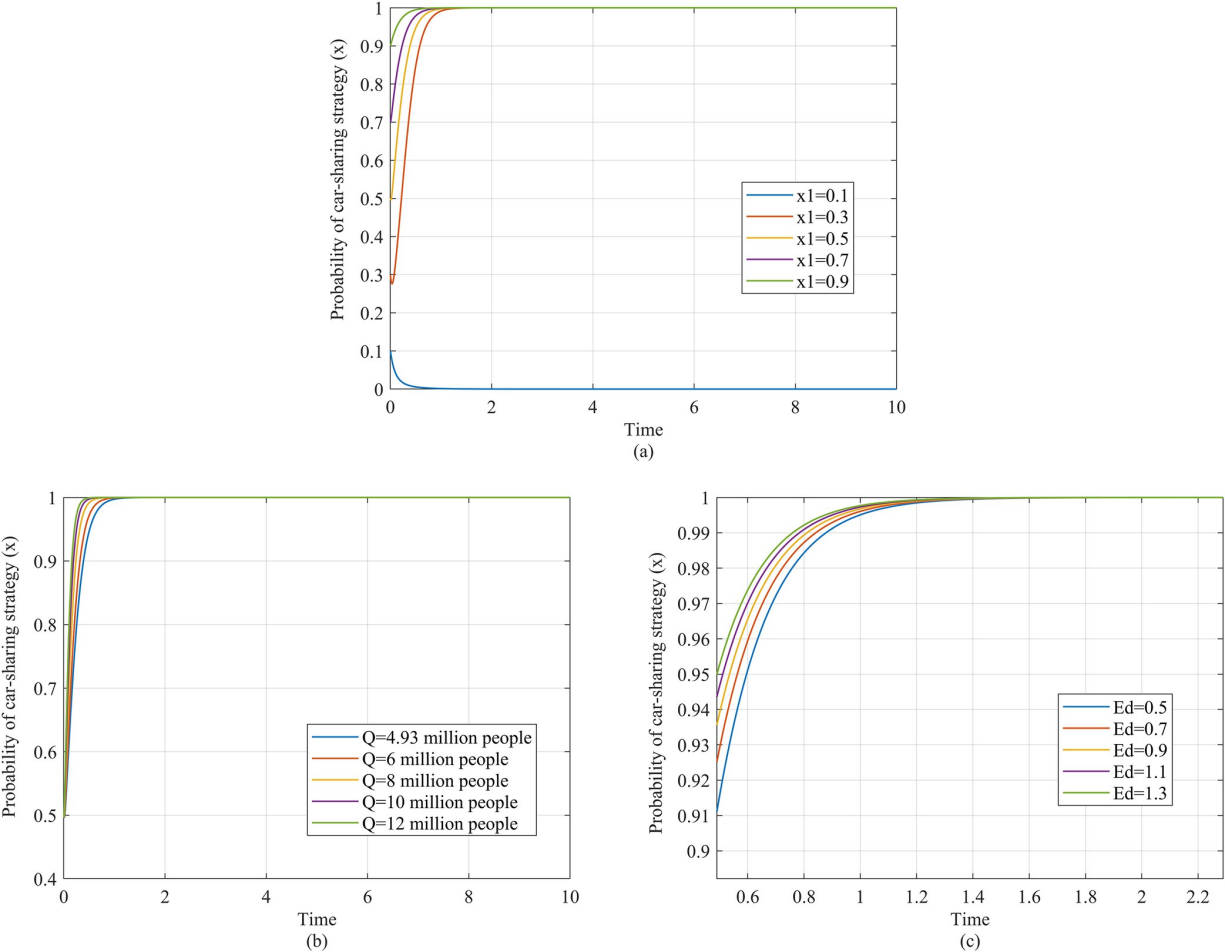
Travelers’ strategy evolution under different initial probability of car-sharing strategy, market potential, and demand elasticity. (a) Description of the travelers’ strategy evolution as initial probability of selecting car-sharing strategy changes. (b) Description of the travelers’ strategy evolution as number of travelers changes. (c) Description of the travelers’ strategy evolution as demand elasticity changes.

**Fig 6:**
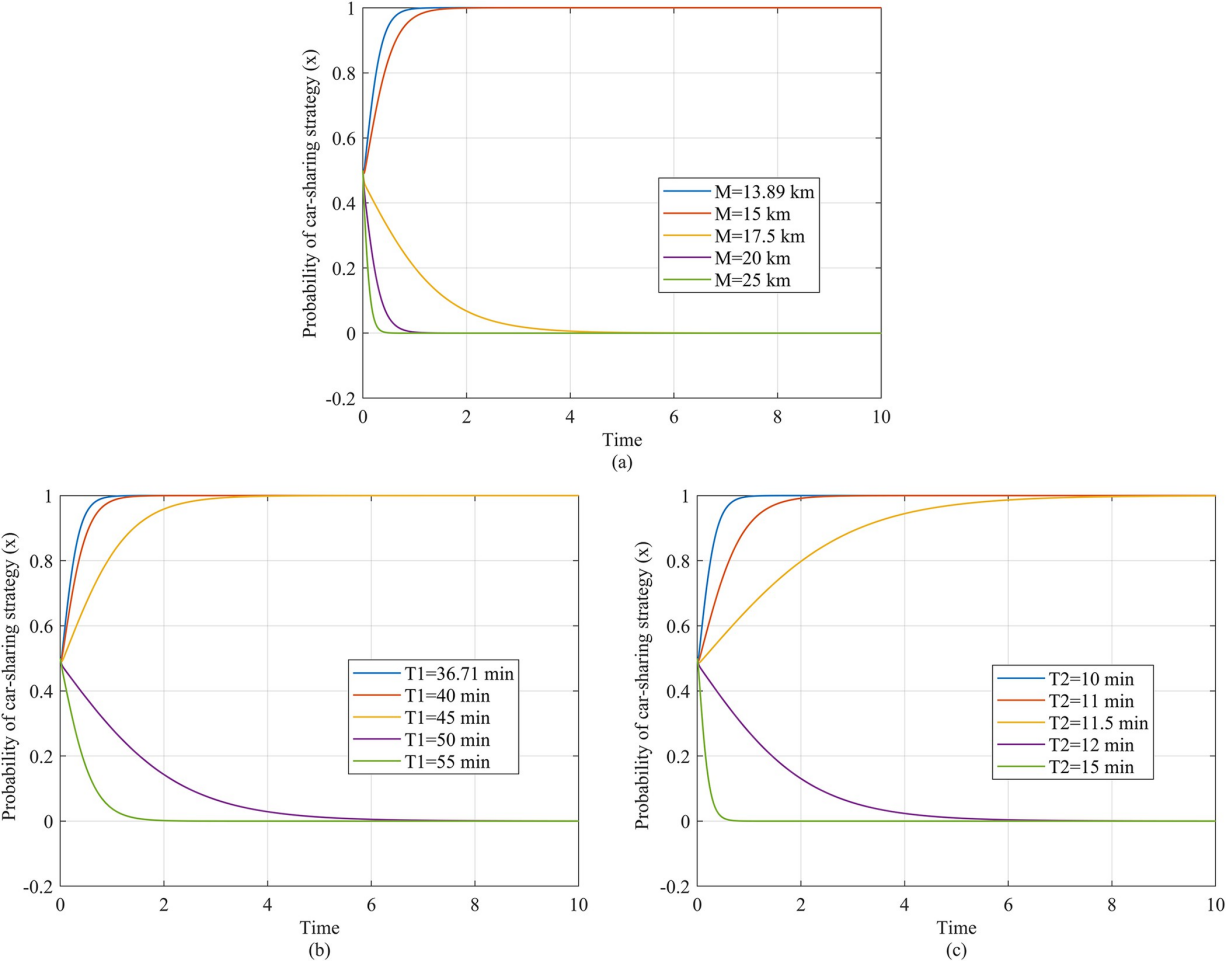
Travelers’ strategy evolution under varying travel mileage, travel duration, and pick up and return time. (a) Description of the travelers’ evolution as travel mileage changes. (b) Description of the travelers’ strategy evolution as travel duration changes. (c) Description of the travelers’ strategy evolution as pick up and return time changes.

Next, in order to explore the influence of different factors, including market conditions and travel characteristics, on the effect of subsidy policies, we simulate the strategic evolution of the traveler as six variables are adjusted, as shown in Figs 5 and 6.

#### 4.2.1 Impact of market conditions on subsidy efficiency

Three variables, the proportion of travelers selecting car-sharing (existing user scale), *x*_1_, the number of travelers (market potential), *Q*, and the demand elasticity for car-sharing, *E*_*d*_, are used to describe market conditions. Fig 5 explores the impact of those three variables on subsidy efficiency.

Based on Fig 5(A), we observe that, as the values of *x*_1_ increases, the willingness of the traveler to select car-sharing also increases. Specifically, when *x*_1_ is 0.1, the traveler maintains the private car strategy, which is shown by the blue curve. When *x*_1_ is 0.3, the travelers’ strategy quickly evolves to car-sharing, as shown by the red curve. As *x*_1_ continues to increase, the transition to the car-sharing strategy occurs more rapidly, as seen by the yellow, purple, and green curves (where *x*_1_ is 0.5, 0.7, and 0.9).

Turning to Fig 5(B), we find that the higher is *Q*, the greater is the travelers’ willingness to select car-sharing. Specifically, as *Q* increases from 4.93 million people to 12 million people, the time taken for the travelers’ evolution to the car-sharing strategy decreases.

From Fig 5(C), it can be seen that, as the demand elasticity gradually increases from 0.5 to 1.3, car-sharing becomes the stable strategy more quickly. This is shown by the fact that the curves corresponding to higher demand elasticity are closer to the upper left in the Fig 5(C), with the green curve (*E*_*d*_ = 1.3) closest to the upper left, and the blue curve (*E*_*d*_ = 0.5) farthest from the upper left.

Fig 5(A) indicates that the higher is the initial proportion of travelers using car-sharing, the higher is the efficiency of the subsidy policy. That is, a traveler is more willing to choose the car-sharing strategy under the same subsidy amount. Since *x*_1_ has no effect on the inequality $y>\frac{Q_{2}}{Q_{1}*(1+\frac{S}{(C_{o}+C_{s}+C_{I})*M+C_{p2}-C_{p1}-P_{m}*M-P_{t}{*T}_{1}{-C}_{t}*T_{2}})-Q_{2}}$, the impact of *x*_1_ on the traveler cannot be explained based on stability theory. Instead, it can be explained by the theory of network externality, which means that the utility of a product increases as the number of users increases, leading consumers to be more willing to use it [77, 78]. The network externality is proportional to the number of users, and the stronger is the product’s network externality, the more that new users will attract other users. Therefore, when government subsidies attract new users to car-sharing, more existing users or a greater network externality means that more additional travelers will be attracted to use car-sharing. Previous studies find that travelers’ willingness to use car-sharing is influenced by whether other travelers are using it [79], which may support the role of network externalities.

Fig 5(B) demonstrates that the more travelers there are, indicating a greater market potential for shared travel, the higher is the efficiency of the subsidy policy. When the government subsidy makes the inequality $y>\frac{Q_{2}}{Q_{1}*(1+\frac{S}{(C_{o}+C_{s}+C_{I})*M+C_{p2}-C_{p1}-P_{m}*M-P_{t}{*T}_{1}{-C}_{t}*T_{2}})-Q_{2}}$ hold, car-sharing becomes the travelers’ stable strategy. From $Q_{1}=Q(1-\frac{{TC}_{CS}-{TC}_{PC}-S}{{TC}_{PC}}E_{d})$ and $Q_{2}=Q(1-\frac{{TC}_{CS}-{TC}_{PC}}{{TC}_{PC}}E_{d})$, it can be seen that, as *Q* increases, the inequality $y>\frac{Q_{2}}{Q_{1}*(1+\frac{S}{(C_{o}+C_{s}+C_{I})*M+C_{p2}-C_{p1}-P_{m}*M-P_{t}{*T}_{1}{-C}_{t}{*T}_{2}})-Q_{2}}$ is more likely to hold, and then the traveler is more willing to choose the car-sharing strategy. This is reflected in the fact that the speed at which the traveler stabilizes at the car-sharing strategy increases with the increase of *Q*. That is, the subsidy efficiency improves.

Fig 5(C) indicates that the higher is the demand elasticity, the higher is the subsidy efficiency. When the subsidy amount is high (such as *S* = 15 CNY per trip), we have *TC*_*CS*_−*TC*_*PC*_−*S*<0, and the traveler’s demand for traveling by car-sharing $Q(1-\frac{{TC}_{CS}-{TC}_{PC}-S}{{TC}_{PC}}E_{d})$ increases as the demand elasticity increases. Therefore, the entire population of travelers will obtain a greater payoff by choosing the car-sharing strategy and travelers will thus be more willing to choose the car-sharing strategy, which shows that the subsidy efficiency increases as the demand elasticity increases.

#### 4.2.2 Impact of travel characteristics on subsidy efficiency

Fig 6 explores the impact of *M* (travel mileage), *T*_1_ (travel time) and *T*_2_ (time taken for picking up and returning vehicle). Based on Fig 6(A), we observe that, as *M* increases, the traveler’s willingness to select the car-sharing strategy decreases, so the subsidy has lower efficiency. Specifically, when *M* is 13.89 km, the traveler retains the car-sharing strategy, but when *M* is 15 km, the time taken for the traveler’s strategy to evolve to car-sharing becomes longer, as shown by the red curves. When *M* is 17.5km, 20 km or 25 km, the traveler’s strategy evolves to using a private car, and the time spent for this evolution decreases as *M* increases.

Based on Fig 6(B), we find that, as *T*_1_ increases, the traveler’s willingness to select the car-sharing strategy decreases, so the subsidy has lower efficiency. Specifically, when *T*_1_ is 36.71 min, the traveler retains the car-sharing strategy, but when *T*_1_ is 40 min or 45 min, the time taken for the evolution to the car-sharing strategy lengthens, as shown by the red and yellow curves. When *T*_1_ is 50 min or 55 min, the traveler’s strategy evolves to using a private car, and the time taken for this decreases as *M* increases.

Based on Fig 6(C), we find that the higher is *T*_2_, the lower is the travelers’ willingness to select car-sharing, and the travelers are highly sensitive to *T*_2_. Specifically, when *T*_2_ is 10 min, travelers continue to follow the car-sharing strategy, when *T*_2_ is 12 min, the travelers’ strategy has already evolved to using a private car, as the purple curve shows, and when *T*_2_ is 15 min, the time taken to evolve to the private car strategy decreases.

Fig 6 indicates that, for travelers with shorter travel mileage, duration, and time taken to pick up and return the vehicle, a subsidy is more effective. According to the payoff of the car-sharing strategy *S*−*P*_*m*_*M*+*P*_*t*_*T*_1_+*C*_*p*1_+*C*_*t*_*T*_2_ and the travel cost of a private car (*C*_*o*_+*C*_*s*_+*C*_*I*_)*M*+*C*_*p*2_, it can be seen that, as *M* increases, if the marginal increase in travel cost of the private car are lower than those for car-sharing, the inequality $y>\frac{Q_{2}}{Q_{1}*(1+\frac{S}{(C_{o}+C_{s}+C_{I})*M+C_{p2}-C_{p1}-P_{m}*M-P_{t}{*T}_{1}{-C}_{t}*T_{2}})-Q_{2}}$ will be more difficult to meet and the traveler will be less likely to choose the car-sharing strategy. When *M* is low, the government subsidy offsets the higher cost of car-sharing compared to private cars, so the traveler chooses the car-sharing strategy. When *T*_1_ or *T*_2_ is low, the subsidy makes the cost of shared travel lower than the cost of using a private car and the traveler chooses the car-sharing strategy. When *T*_1_ or *T*_2_ is high, the cost of shared travel is greater, the subsidy does not make the cost of shared travel lower than that of using a private car, and the traveler will choose the private car strategy.

We also conducted a sensitivity analysis to check the robustness of the results, which is presented in S2 Appendix.

### 4.3 Strategic evolution of players under other subsidy mode

**Fig 7:**
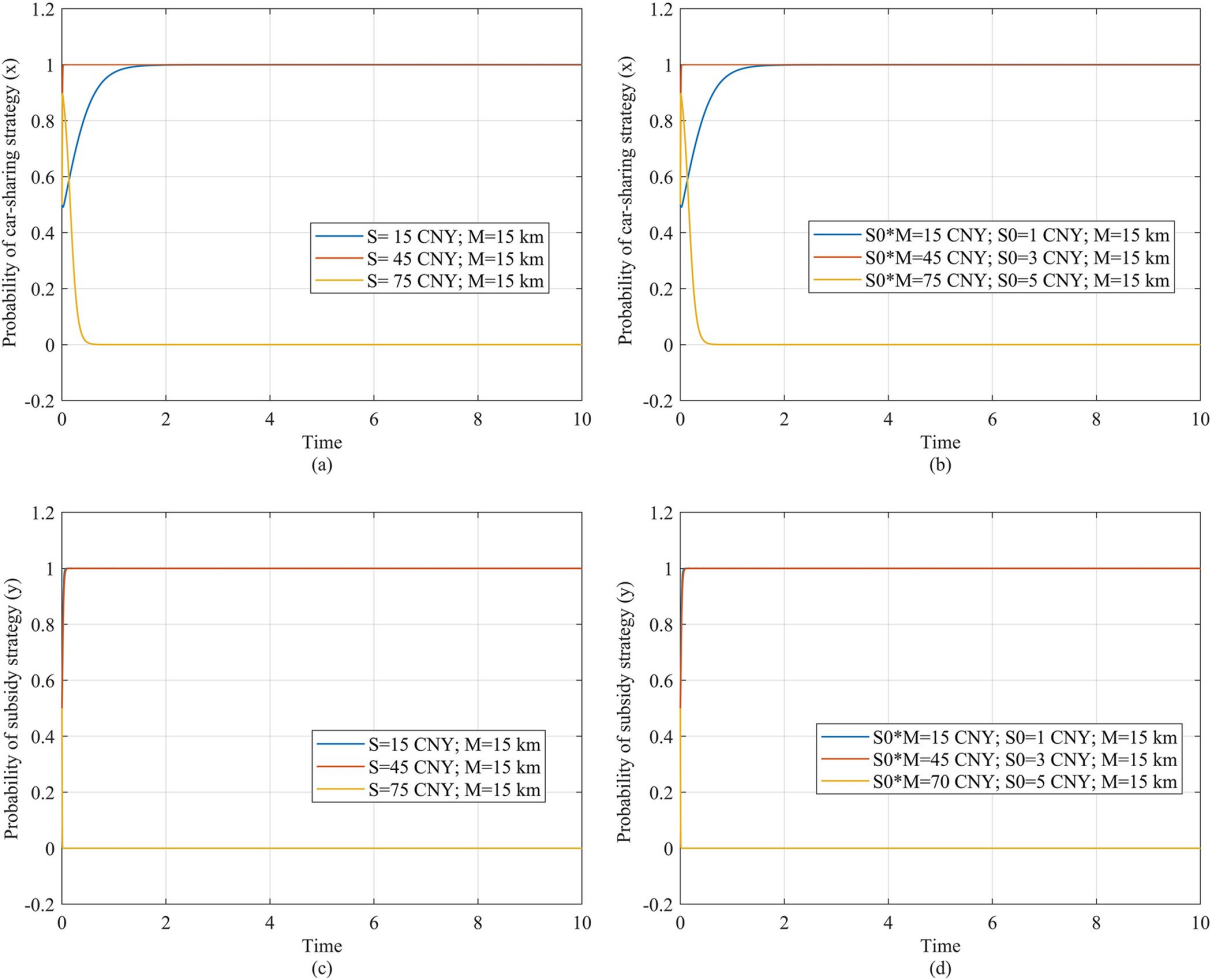
Travelers’ strategy evolution under two subsidy modes. (a) Description of the travelers’ strategic evolution under fixed subsidy. (b) Description of the travelers’ strategy evolution under mileage-based subsidy.

In addition to the fixed subsidy, we also simulate the strategy selection of the traveler under a mileage-based subsidy and compare it with the fixed subsidy to determine the better subsidy mode. The mileage-based subsidy can be represented by *S*_0_*M*, where *S*_0_ represents the subsidy provided for each kilometer traveled. Fig 7 compares the effects of the mileage-based subsidy and the fixed subsidy. We make the subsidy cost under the two subsidy models the same, and keep the other parameters unchanged. Fig 7(B) and 7(B) show the travelers’ strategy evolution under the fixed subsidy and mileage-based subsidy respectively, and Fig 7(C) and 7(D) show the government’s strategy evolution. It can be seen that, under the same subsidy cost, the subsidy effects on players under the two modes remain consistent.

**Fig 8:**
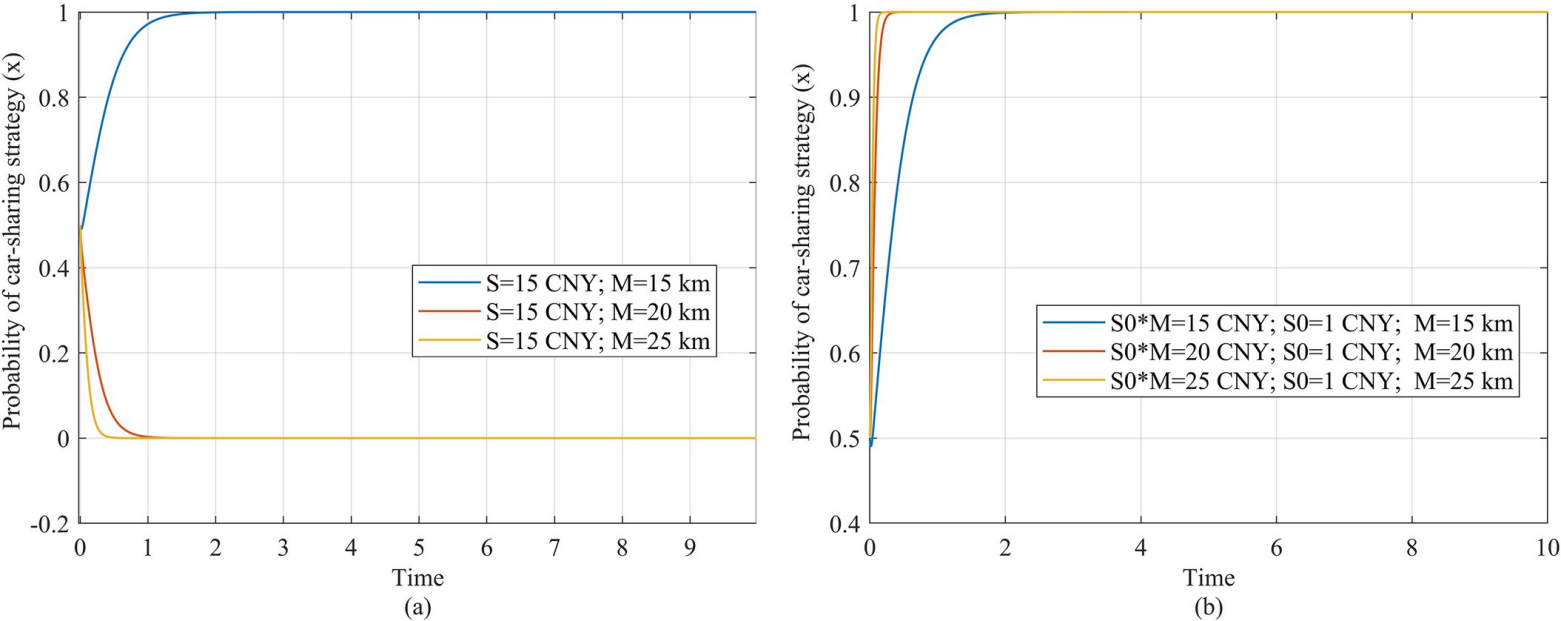
Government’s strategy evolution under two subsidy modes. (a) Description of the government’s strategic evolution under fixed subsidy. (b) Description of the government’s strategy evolution under mileage-based subsidy.

Next, we simulate the impact of travel mileage on subsidy efficiency under the mileage-based subsidy mode, and investigate the differences between the two subsidy modes. Under the fixed subsidy, as the travel mileage increases, there is lower subsidy efficiency, and the traveler is less willing to choose car-sharing (Fig 8(A)). However, under the mileage-based mode, the willingness to use shared travel increases with the increase in travel mileage, thus there is higher subsidy efficiency. In Fig 8(B), as the travel mileage increases from 15 km to 75 km, the traveler evolves to the car-sharing strategy more quickly.

The reason for this difference is that, under the mileage-based subsidy, as the traveler’s mileage increases, the subsidy benefits and travel costs will also increase. If the increase in the subsidy benefits *S*_0_*M* is higher than the increase in the travel costs *P*_*m*_*M*, the traveler will have a stronger motivation to choose the car-sharing strategy. Meanwhile, as the travel mileage increases, if the increase in the subsidy cost *S*_0_*M* for the government is less than the increase in environmental benefits *BM*, the government will keep the subsidy strategy, providing subsidy benefits *S*_0_*M* to the traveler. Under the fixed subsidy, as the travel mileage increases, the travel costs of car-sharing *P*_*m*_*M* increase, while the subsidy benefits *S* remain unchanged, so the car-sharing strategy’s payoff (*S*−*P*_*m*_*M*−*P*_*t*_*T*_1_−*C*_*p*1_−*C*_*t*_*T*_2_) decreases and the traveler’s willingness to choose the car-sharing strategy also decreases.

In addition, we simulated the impact of another five variables on subsidy efficiency, under the mileage-based subsidy, which can be seen in S3 Appendix. We found similar results to those for the fixed subsidy.

## 5. Discussion

This paper constructs an evolutionary game model including travelers and the government, simulates the strategic evolution of the two players under a subsidy policy, and explores the impacts of different variables on the subsidy policy. Next, we will discuss the simulation results.

### 5.1 Impact of subsidy policies on travelers and government

The research results indicate that a subsidy threshold exists. When the subsidy exceeds the threshold, the government chooses the non-subsidy strategy due to the excessive subsidy costs. Consequently, without the subsidy, the traveler’s cost of car-sharing increases and they choose the private car strategy. This implies that the subsidy needs to be kept below this threshold to enable the government to sustain the subsidy strategy, while the subsidy still needs to be high enough to encourage the traveler to choose the car-sharing strategy. Specifically, the government subsidy should be lower than the environmental benefit brought about by shared travel, and higher than the difference in travel costs between the private car and car-sharing. Furthermore, given the varying socio-economic conditions across different regions, the subsidy amount must be tailored to local conditions. Local governments should enhance their cooperation with car-sharing operators to obtain support with data and to hear about their experiences, which would enable a thorough evaluation of the benefits and costs of a subsidy policy in the local context so that the appropriate subsidy amount can be determined. Previous research has not paid enough attention to the government’s subsidy costs and has insufficiently explored the optimal subsidy amounts for car-sharing users, making such policies potentially unsustainable. The findings of this study provide a reference for the design of subsidy amounts.

### 5.2 Impact of different variables on subsidy efficiency

#### 5.2.1 Discussion of mileage, duration, and pick up and return time

We find that the impact of travel mileage on subsidy efficiency varies under different subsidy modes. Under a fixed subsidy, subsidy efficiency increases with a decrease in travel mileage, while under the mileage-based subsidy, subsidy efficiency increases with an increase in travel mileage. The reason is that the mileage-based subsidy reverses the situation wherein the travel cost advantages of car-sharing are mainly seen in short-mileage travel [51–53]. For example, based on data from Lisbon, Martin et al. [51] found that, compared to private cars, car-sharing costs less when it comes to short-mileage travel. Unlike existing research, this paper finds that with a mileage-based subsidy, car-sharing can also demonstrate travel cost advantages for long-mileage travel, and such a subsidy would be sustainable. This result can help with the design of more comprehensive and targeted subsidy policies to encourage shared travel. Our result suggests that the two subsidy modes are applicable to travelers with different travel characteristics, which indicates that the government should adopt differentiated subsidy policies. Specifically, when the travel mileage is low, using fixed subsidies encourages shared travel, while when the travel mileage is high, using mileage-based subsidies encourages shared travel. In practice, the government could collaborate with car-sharing operators to launch two types of car-sharing coupons. The first would be applicable to travel below a certain mileage (the mileage values should be determined based on the specific situation in a city or region), and could be used by travelers to offset a fixed amount of the service fees. The second would be applicable to longer-mileage travel, and would offset the service fees based on the travel mileage.

We also find, in both subsidy modes, that when the travel duration is higher, traveling by car-sharing will lead to higher service fees, making travelers less willing to use car-sharing and meaning that subsidies have less impact on users. Previous studies have similarly found that car-sharing has a cost advantage for short-duration trips, and thus government subsidies should focus on short-duration travel [5, 28]. However, we argue that this would not be a suitable subsidy policy. The reason is that focusing on short-duration trips might encourage travelers to drive faster to reduce their travel time, thereby increasing the risk of traffic accidents. Therefore, the government should encourage car-sharing operators to adjust the pricing rules by reducing the weight of time-based charges and increasing the weight of mileage-based charges. This adjustment would decrease the negative impact of travel duration on subsidy efficiency, simultaneously reducing the accident risks.

In addition, under the two subsidy modes, the greater is the time taken to pick up and return a vehicle in car-sharing, the higher is the opportunity cost for travelers of using car-sharing, and the lower the subsidy efficiency. This indicates the importance of shortening the time required for the pick-up and return. The government can encourage travelers to use other travel modes to shorten the pick-up and return time through another subsidy, such as collaborating with car-sharing operators and bike-sharing operators to launch a combined service that offers preferential rates.

**5.2.2 Discussion of user scale, market potential, and elasticity**We found that, under the two subsidy models, as the initial probability of travelers choosing car-sharing (existing user scale) increased, the subsidy efficiency increased. As mentioned earlier, this result can be explained by the theory of network externalities, which states that the demand for a product increases with the number of users [78]. Based on this result and its theoretical explanation, the government should adopt different subsidy levels according to the current number of users of car-sharing. Specifically, the government should use higher subsidies to encourage the use of car-sharing when there are few existing users, but as the number increases, and network externalities are developed, this will allow the government to gradually reduce the subsidies.

We also found that an increase in the number of travelers (market potential) or the demand elasticity for car-sharing would make the subsidy efficiency higher. This finding suggests that subsidy policies should be prioritized in regions with high potential and high demand elasticity in the car-sharing market to improve the efficiency of the subsidy. According to the elasticity theory, when the market potential is greater, the personal characteristics of the travelers may become more diverse. Therefore, the overall demand elasticity of travelers in the market will be higher [80]. This theory suggests that regions with higher market potential also have higher demand elasticity, and therefore market potential is a key factor when designing subsidy policies. Specifically, subsidy policies should be prioritized for cities or regions with more potential car-sharing travelers, such as metropolitan areas, which often have a high market potential for car-sharing [81]. In addition, in cities or regions with limited market potential, encouraging the use of alternative travel modes such as public transportation may be a better way to replace private cars.

When exploring subsidy policies for car-sharing users, the existing literature has considered market conditions in models, such as the number of travelers and demand elasticity [24], but their impact has not been fully analyzed. Compared to previous studies, this paper explores the impact of market conditions on subsidy efficiency, and the corresponding findings are of great significance for analyzing the market behavior of car-sharing and designing subsidy policies.

## 6. Conclusions

Car-sharing is a more environmentally friendly travel mode, as it has the potential to reduce urban pollution by replacing private cars. However, a lack of willingness among travelers to adopt car-sharing as their preferred travel mode is a challenge. Therefore, it is necessary to encourage car-sharing and one potential way to achieve this is through government subsidy policies. This paper introduces an evolutionary game model, including travelers and the government, by simulating the strategic evolution of players as subsidy values and other variables are altered. The paper explores possible subsidy principles to more effectively encourage travelers’ adoption of car-sharing.

### 6.1 Research conclusions

Firstly, the amount of government subsidies should be lower than the environmental benefits brought about by shared travel, and higher than the difference in travel costs between private cars and car-sharing, in order to ensure the effectiveness and sustainability of subsidy policies. Secondly, under a fixed subsidy, the subsidy efficiency increases with a decrease in travel mileage, while under the mileage subsidy model, the opposite is true. Therefore, when the travel mileage is low, a fixed subsidy should be adopted to encourage shared travel, but when the travel mileage is high, mileage-based subsidies should be adopted. Thirdly, under the two subsidy modes, the subsidy efficiency decreases with increases in travel duration and the pick-up and return time. The government should subsidize travelers to help them shorten their pick-up and return times using other travel modes such as bike-sharing, and should encourage operators to reduce their time-based service fees to optimize their charging modes. Fourthly, under the two subsidy modes, the more existing users there are, the higher the market potential, and the higher demand elasticity, the higher will be the subsidy efficiency. The government should increase subsidies to expand the number of car-sharing users, and prioritize implementing subsidy policies in areas with a large number of potential car-sharing travelers to improve subsidy efficiency.

### 6.2 Limitations and future work

This paper explores the principles of subsidies to more effectively promote the use of car-sharing, which would be conducive to mitigating urban pollution. However, it has the following limitations. Firstly, in the paper, we only consider two players, travelers and the government, in the game model, without considering other game players such as car-sharing operators. In fact, the government could subsidize car-sharing operators to reduce service fees and improve service quality so as to attract more travelers to use car-sharing. In the future, we will include operators as a game player to explore the impact of their strategy selection on subsidy efficiency and sustainability, and thereby draw richer and deeper conclusions. Secondly, the conclusions of this paper are based on data from Beijing. Whether the conclusions in the paper are sufficiently robust when applied to other countries or cities needs further exploration. In the future, we will focus on collecting data from other countries or cities to carry out more extensive research and verify the robustness of our conclusions.

## Supporting information

S1 Appendix Data and code. (DOCX)

S2 Appendix Sensitivity analysis. (DOCX)

S3 Appendix Strategy evolution of travelers under mileage-based subsidy mode. (DOCX)

S1 Fig Calculation program and steps. (TIF)

S2 Fig Sensitivity analysis of Section 4.1’s results. (TIF)

S3 Fig Sensitivity analysis of Section 4.2.1’s results. (TIF)

S4 Fig Sensitivity analysis of Section 4.2.2’s results. (TIF)

S5 Fig Traveler’s strategy evolution under mileage-based subsidy. (TIF)

S6 Fig Traveler’s strategy evolution under fixed subsidy. (TIF)
