# Supplementary material for: How to design subsidy policies to better encourage travelers to use car-sharing instead of private cars? An evolutionary game study
Source: PLoS One. 2024 Sep 19;19(9):e0308622. doi: 10.1371/journal.pone.0308622 (PMC11412671; doi:10.1371/journal.pone.0308622)
Supplement: S1 Appendix — (DOCX) [file pone.0308622.s001.docx]

S1 Appendix

**Data and its source (Beijing, 2022)**

| **Meaning** | **Value** | **Unit** | **Source** |
| --- | --- | --- | --- |
| Average travel mileage | 13.89 | Km | A |
| Average travel duration | 36.71 | Min | A |
| Average parking cost of car-sharing | 1.25 | CNY /one trip | A |
| Average parking cost of private cars | 3.19 | CNY/one trip | A |
| Average maintenance cost of private cars | 0.26 | CNY/km | A |
| Average insurance cost of private cars | 0.47 | CNY/km | A |
| Number of private cars | 4930000 | Vehicle | A |
| Rental fee (mileage) | 1.5 | CNY/km | B |
| Rental fee (time) | 0.1 | CNY/min | B |
| Per capita per minute income of citizens | 0.67 | CNY/min | C |
| Average fuel consumption of private cars | 0.05 | Liter/km | D |
| Fuel price | 7.58 | CNY/ liter | E |
| Average reduction in CO2 emissions of car-sharing | 114.9 | g/km | F |
| Average carbon price | 0.03 | CNY/g | G |

A:Data comes from Beijing Transportation Development Research Institute. <https://www.bjtrc.org.cn/List/index/cid/7/p/1.html>

B:Data comes from GoFun.

[https://www.shouqiev.com/travel](file:///C:\Users\Hasee\Desktop\%20https:\www.shouqiev.com\travel)

C:Data comes from Beijing Statistical Yearbook.

<http://tjj.beijing.gov.cn/tjsj_31433/>

D:Data comes from Ministry of Industry and Information Technology of the People’s Republic of China. <https://wap.miit.gov.cn/zwgk/zcwj/wjfb/gg/art/2022/art_02d2d195cc5344d498f3a4faf508054f.html#:~:text=2021%E5%B9%B4%E5%BA%A6%EF%BC%8C%E4%B8%AD,79.79%E4%B8%87%E5%88%86%E3%80%82>

E:Data comes from Oil Price Information Web. <https://quote.cngold.org/oil/youjia_tzsjb2023.html>

F:Data comes from China Automotive Technology&Research Center Co., Ltd.

<https://www.catarc.ac.cn/>

G:Data comes from Beijing Carbon Emissions Trading Platform.

<https://www.bjets.com.cn/article/scyj/tscbb/>

The numerical simulation program and its operational steps are listed in S1 Fig.

**S1** **Fig. Calculation program and steps.** Description of how numerical simulation is carried out in MATLAB.
