# Supplementary material for: How to design subsidy policies to better encourage travelers to use car-sharing instead of private cars? An evolutionary game study
Source: PLoS One. 2024 Sep 19;19(9):e0308622. doi: 10.1371/journal.pone.0308622 (PMC11412671; doi:10.1371/journal.pone.0308622)
Supplement: S2 Appendix — (DOCX) [file pone.0308622.s002.docx]

S2 Appendix

We conducted a sensitivity analysis of Section 4.1’s simulation results. We simulated the impact of subsidy $S$ on the players under different values of $x_{1}$, $y_{1}$ and $\delta$. We adjusted the initial probability of selecting car-sharing $x_{1}$ and the subsidy strategy $y_{1}$ from 0.5 to 0.2 and 0.8, and the values of the coefficient $\delta$ from 5 to 2 and 10. The simulation results are shown in S2 Fig. From S2 Fig, it can be seen that there is still a threshold. When subsidies are below the threshold, the travelers’ willingness to use car-sharing and the government’s willingness to subsidize the travelers increase with a rising subsidy amount. When subsidies are higher than the threshold, the travelers’ willingness to use car-sharing and the government’s willingness to subsidize the travelers decrease with a rising subsidy amount. Therefore, the simulation results are consistent with the results in Section 4.1.

**S2 Fig. Sensitivity analysis of Section 4.1’s results.** (a), (b) Description of travelers’ strategy evolution under changing $S$ and different $x_{1}$, $y_{1}$ and $\delta$. (c), (d) Description of government’s strategy evolution under changing $S$ and different $x_{1}$, $y_{1}$ and $\delta$.

Similarly, we conducted a sensitivity analysis of the results in Section 4.2.1. S3 Fig shows the results regarding variables $x_{1}$, $Q$ and $E_{d}$. We simulated the impact of $x_{1}$ $Q$ and $E_{d}$ on the subsidy efficiency under different values of $S$, $x_{1}$, $y_{1}$ and $\delta$. We adjusted the initial probability of selecting car-sharing $x_{1}$ and selecting the subsidy strategy $y_{1}$ from 0.5 to 0.2 and 0.8, the value of the coefficient $\delta$ from 5 to 2 and 10, and the value of the subsidy $S$ from 15 CNY per trip to 10 CNY per trip and 20 CNY per trip. The simulation results are shown in Fig B2. From S3 Fig, it can be seen that the higher are the values of $x_{1}$, $Q$ and $E_{d}$, the stronger is the travelers’ willingness to use car-sharing. Therefore, the simulation results are consistent with the results in Section 4.2.1.

**S3 Fig. Sensitivity analysis of Section 4.2.1’s results.** (a), (b) Description of travelers’ strategic evolution under changing $x_{1}$, and different $S$, $y_{1}$ and $\delta$. (c), (d) Description of travelers’ strategic evolution under changing $Q$, and different $S$, $x_{1}$, $y_{1}$ and $\delta$. (e), (f) Description of travelers’ strategy evolution under changing $E_{d}$ and different $S$, $x_{1}$, $y_{1}$ and $\delta$.

S4 Fig shows the test results for the rest of the variables. We simulated the impact of travel mileage $M$, travel duration $T_{1}$ and time taken to pick up and return the vehicle $T_{2}$ on the subsidy efficiency under different values of $S$, $x_{1}$, $y_{1}$ and $\delta$. We adjusted the initial probability of selecting car-sharing $x_{1}$ and selecting the subsidy strategy $y_{1}$ from 0.5 to 0.2 and 0.8, the value of the coefficient $\delta$ from 5 to 2 and 10, and the value of the subsidy $S$ from 15 CNY per trip to 10 and 20 CNY per trip. From S4 Fig, it can be seen that the higher are the values of $M$, $T_{1}$ and $T_{2}$, the lower is the travelers’ willingness to use car-sharing. Therefore, the simulation results are consistent with the results in Section 4.2.2.

**S4 Fig. Sensitivity analysis of Section 4.2.2’s results.** (a), (b) Description of travelers’ strategy evolution under changing $M$ and different $S$, $x_{1}$, $y_{1}$ and $\delta$. (c), (d) Description of travelers’ strategy evolution under changing $T_{1}$ and different $S$, $x_{1}$, $y_{1}$ and $\delta$. (e), (f) Description of travelers’ strategy evolution under changing $T_{2}$ and different $S$, $x_{1}$, $y_{1}$ and $\delta$.
