# Supplementary material for: How to design subsidy policies to better encourage travelers to use car-sharing instead of private cars? An evolutionary game study
Source: PLoS One. 2024 Sep 19;19(9):e0308622. doi: 10.1371/journal.pone.0308622 (PMC11412671; doi:10.1371/journal.pone.0308622)
Supplement: S3 Appendix — (DOCX) [file pone.0308622.s003.docx]

S3 Appendix

We simulated the impact of five variables (travel duration, pick-up and return time, existing user scale, market potential, and demand elasticity) on subsidy efficiency under a mileage-based subsidy. We found that, with the same subsidy cost, the five variables respectively have the same impact on subsidy efficiency under the two subsidy modes. The simulation results for the mileage-based subsidy and fixed subsidy are shown in S5 and S6 Figs respectively, where $M=15$, $S=15$, $S_{0}=1$.

**S5 Fig. Traveler’s strategy evolution under mileage-based subsidy.** (a) Description of traveler’s strategy evolution under different travel duration. (b) Description of traveler’s strategy evolution under different pick up and return time. (c) Description of traveler’s strategy evolution under different user scale. (d) Description of traveler’s strategy evolution under different number of travelers. (e) Description of traveler’s strategy evolution under different demand elasticity.

**S6 Fig. Traveler’s strategy evolution under fixed subsidy.** (a) Description of travelers’ strategy evolution under different travel duration. (b) Description of traveler’s strategy evolution under different pick up and return time. (c) Description of traveler’s strategy evolution under different user scale. (d) Description of traveler’s strategy evolution under different number of travelers. (e) Description of travelers’ strategy evolution under different demand elasticity.

In S5 Fig(a), the speed and trend with which the traveler evolves towards the car-sharing strategy are consistent with S6 Fig (a), indicating that, under the two subsidy modes, the impact of travel duration on subsidy efficiency is the same. Similarly, the simulation results in S5 Figs (b)-(e) are consistent with those in S6 Figs (b)-(e). Therefore, when the subsidy amount remains unchanged, the impact of the five variables on subsidy efficiency is consistent under the two subsidy modes.
