# Supplementary figures and images for: How to design subsidy policies to better encourage travelers to use car-sharing instead of private cars? An evolutionary game study
Source: PLoS One. 2024 Sep 19;19(9):e0308622. doi: 10.1371/journal.pone.0308622 (PMC11412671; doi:10.1371/journal.pone.0308622)

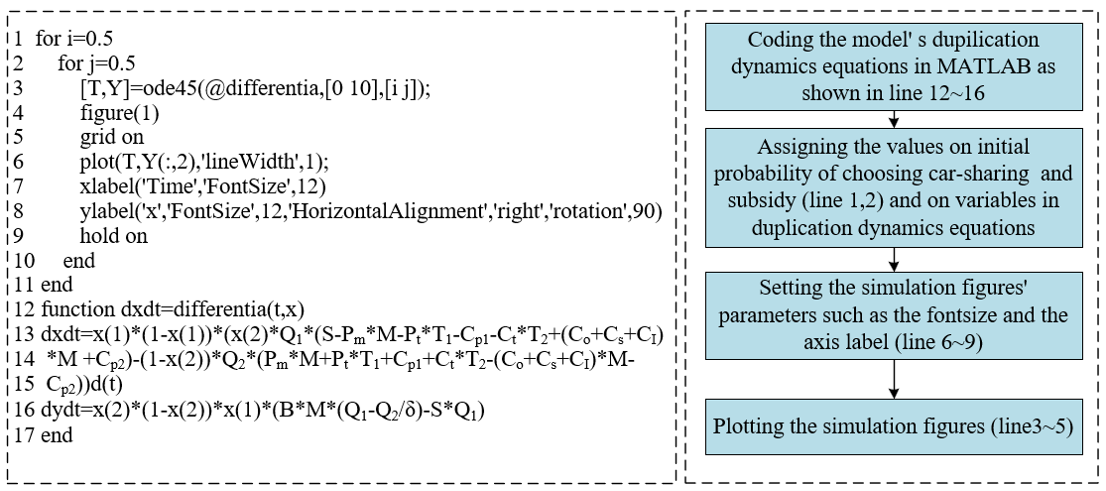

Supplement: S1 Fig — (TIF) [file pone.0308622.s004.tif]

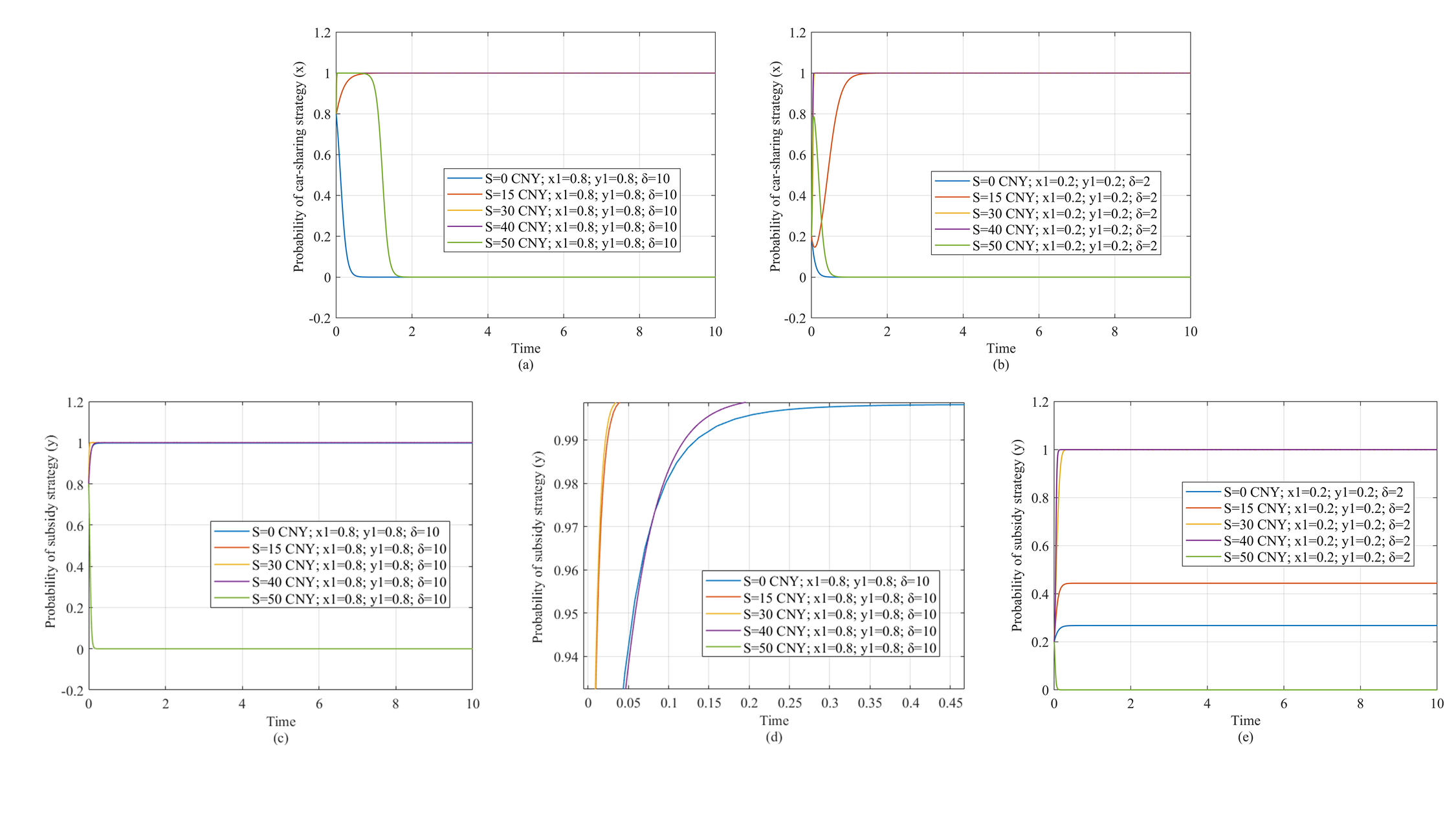

Supplement: S2 Fig — (TIF) [file pone.0308622.s005.tif]

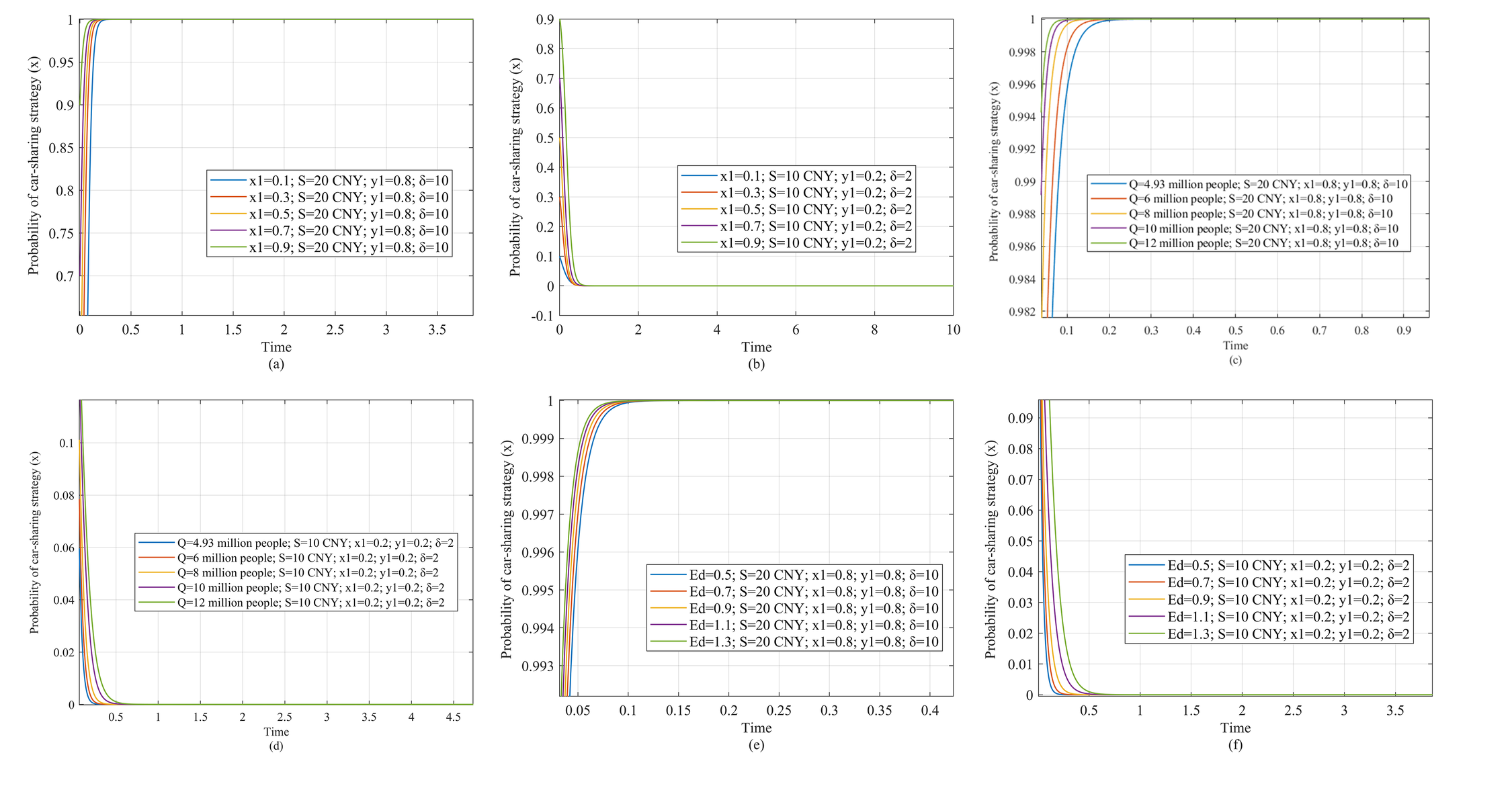

Supplement: S3 Fig — (TIF) [file pone.0308622.s006.tif]

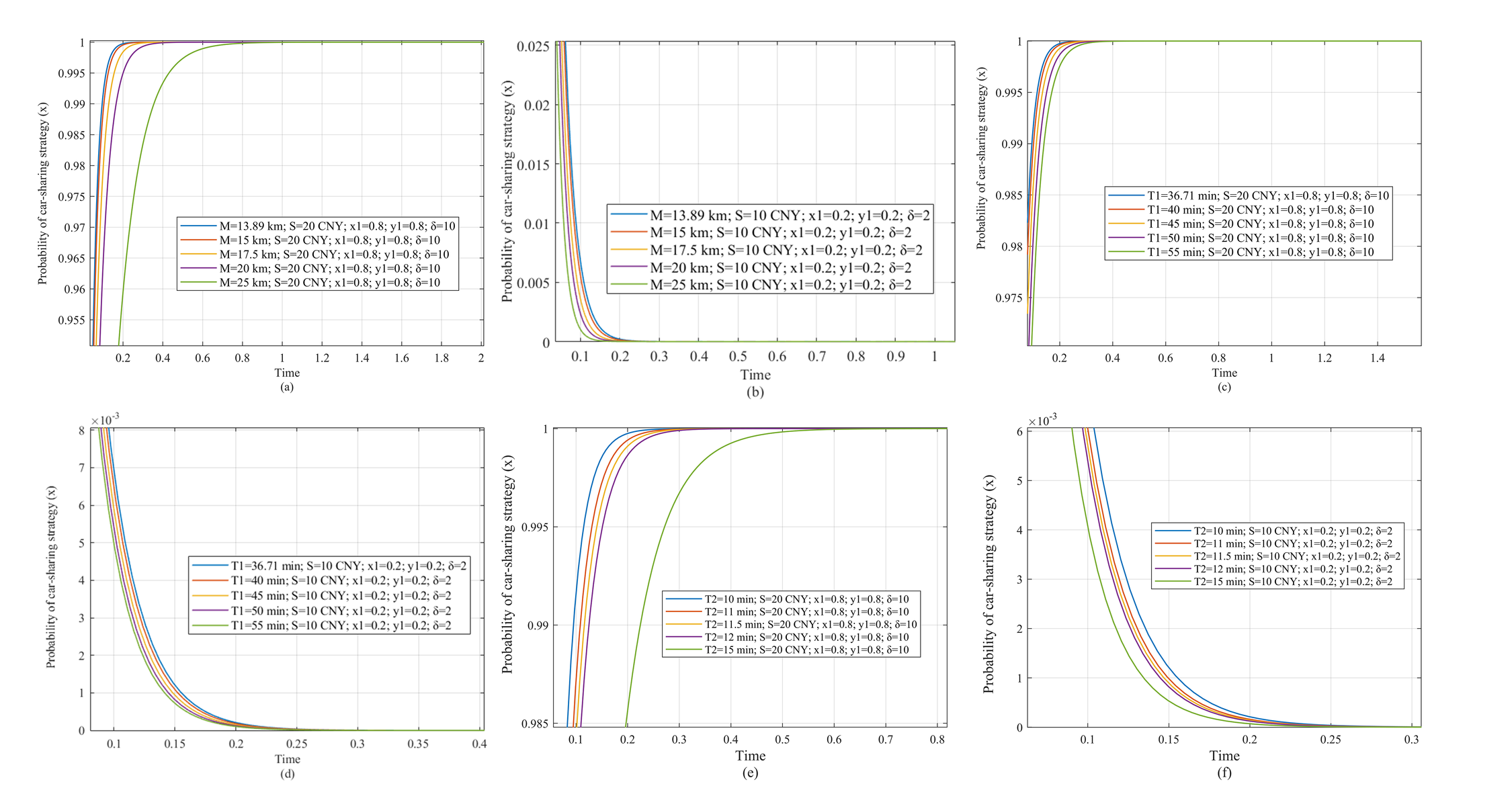

Supplement: S4 Fig — (TIF) [file pone.0308622.s007.tif]

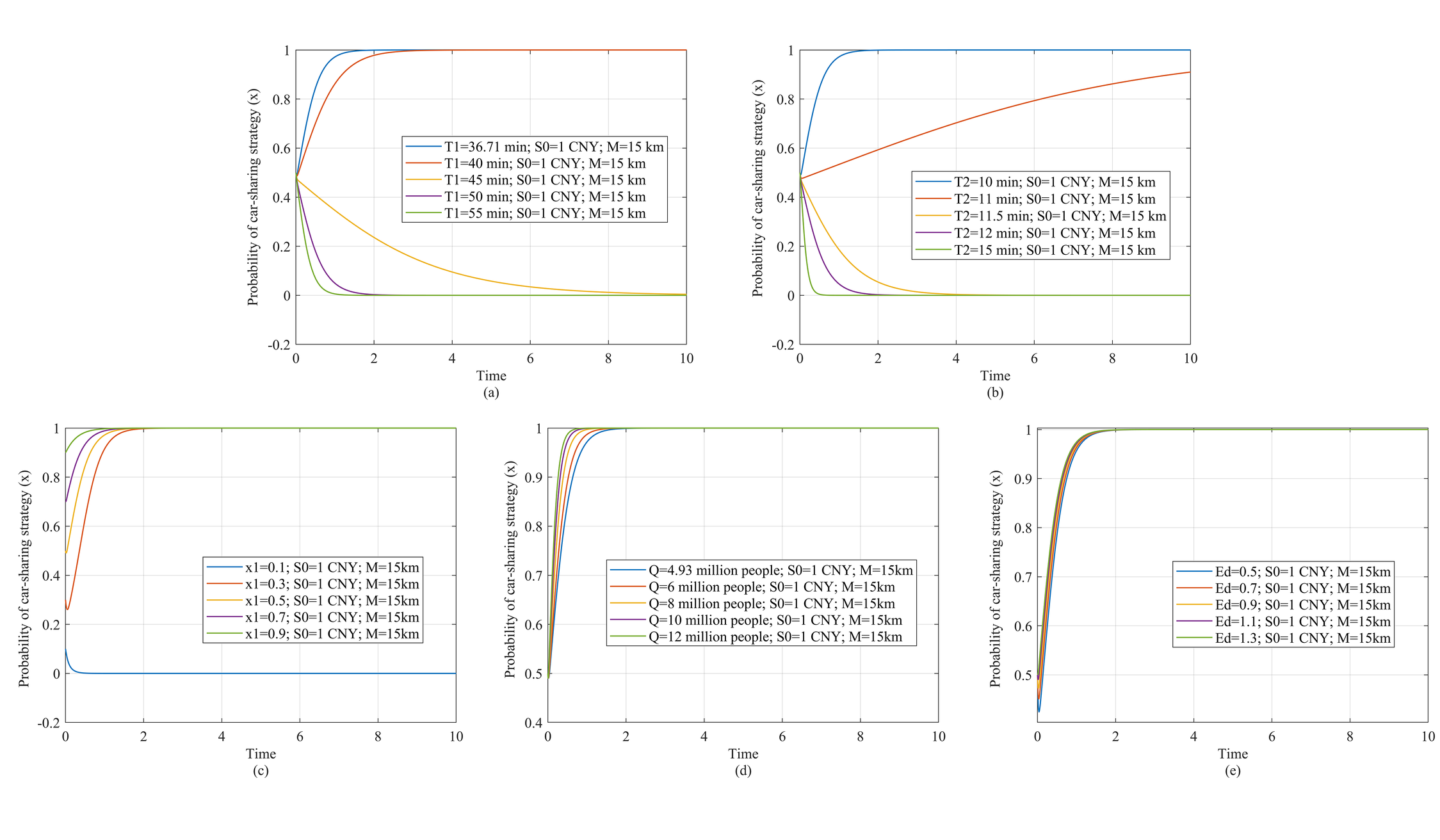

Supplement: S5 Fig — (TIF) [file pone.0308622.s008.tif]

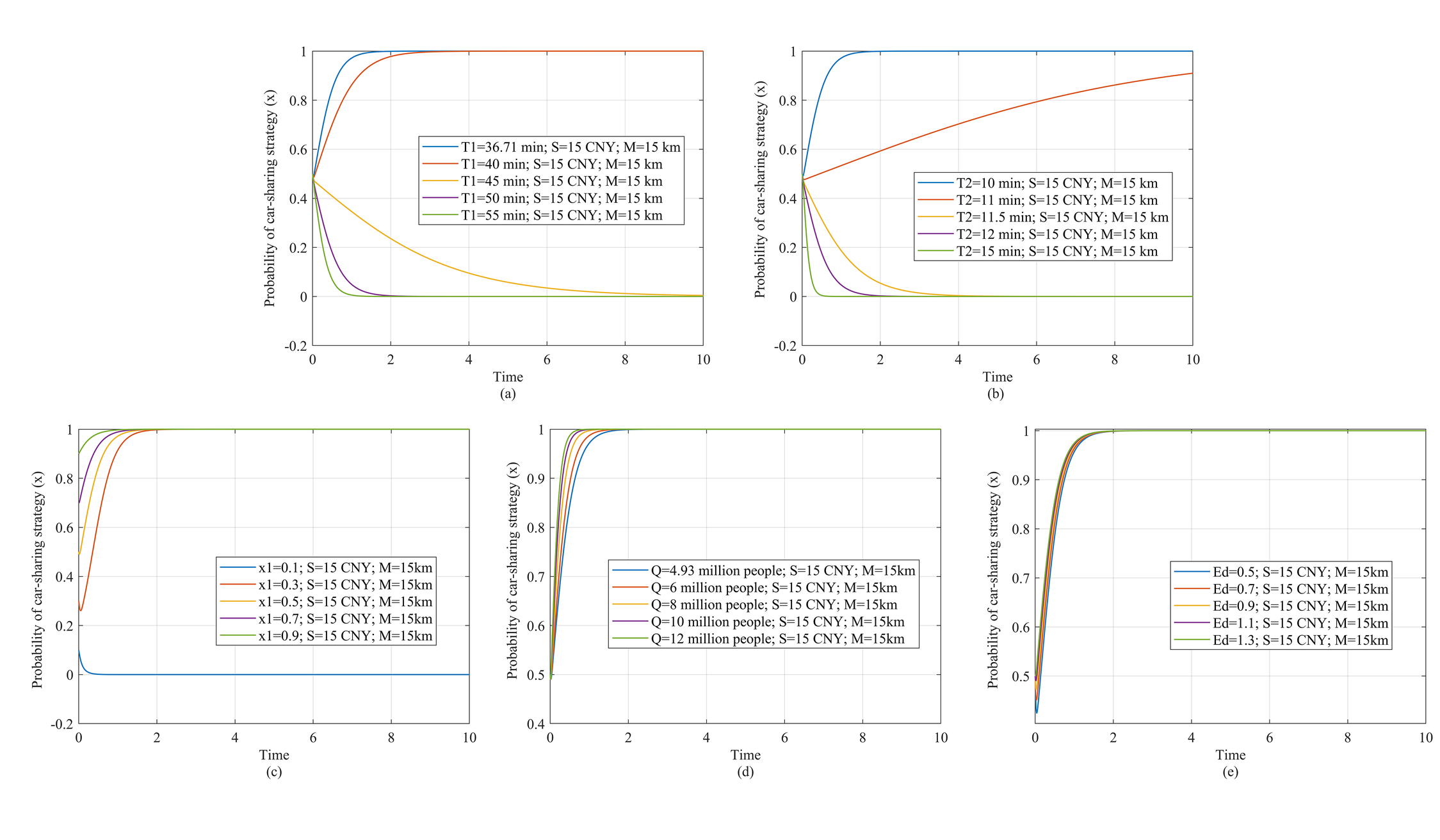

Supplement: S6 Fig — (TIF) [file pone.0308622.s009.tif]
